# Bee Pollen Phytochemicals and Nutrients as Unequaled Pool of Epigenetic Regulators: Implications for Age-Related Diseases

**DOI:** 10.3390/foods14030347

**Published:** 2025-01-21

**Authors:** Rachid Kacemi, Maria G. Campos

**Affiliations:** 1 Observatory of Drug-Herb Interactions, Faculty of Pharmacy, University of Coimbra, Heath Sciences Campus, Azinhaga de Santa Comba, 3000-548 Coimbra, Portugal; 2 Coimbra Chemistry Centre (CQC, FCT Unit 313) (FCTUC), University of Coimbra, Rua Larga, 3004-531 Coimbra, Portugal

**Keywords:** bee pollen, epigenetic regulation, pollen extracellular vesicles, polyphenols, nutrients, aging, DNA, histones, non-coding RNA

## Abstract

Bee pollen is characterized by an exceptional diversity and abundance of micronutrients and bioactive phytochemicals. This richness remains very sparsely investigated, but accumulating evidence strongly supports a promising future for bee pollen in human nutrition and medicine. Epigenetic regulation is among the most compelling biomedical topics that remain completely untapped in bee pollen and bee derivative research. In our current research, we identified numerous ubiquitous compounds that are consistently present in this matrix, regardless of its botanical and geographical origins, and that have been well studied and documented as epigenetic regulators in recent years. Given the relative newness of both bee pollen biomedical research and epigenetic studies within nutritional, pharmaceutical, and medical sciences, this review aims to bridge these valuable fields and advance related experimental investigations. To the best of our knowledge, this is the first work that has aimed to comprehensively investigate the epigenetic modulatory potential of bee pollen compounds. Our findings have also unveiled several intriguing phenomena, such as a dual effect of the same compound depending on the cellular context or the effect of some compounds on the cross-generational heritability of epigenetic traits. Although experimental studies of epigenetic regulation by bee pollen as a whole or by its extract are still lacking, our current study clearly indicates that this research avenue is very promising and worth further investigations. We hope that our current work constitutes a foundational cornerstone of future investigations for this avenue of research.

## 1. Introduction

Aging is a complex, gradual, and innate process of living organisms. In humans, aging is linked to a series of non-communicable diseases that currently constitute the major burden of global healthcare. Neurodegenerative diseases (NDDs) and cancers are among the most challenging ones as they remain insufficiently understood and manageable, but others such as cardiovascular and metabolic diseases remain also very burdensome, even if their understanding and management are much more advanced. In the quest to figure out efficient and safe agents to prevent and treat these diseases, natural compounds occupy a prominent place as it has become widely known in recent years. Bee products, especially bee pollen (BP), are among the most recently coveted natural sources in such a quest. Research conducted so far showed that these products are very rich and safe resources of natural candidates for nutritional and pharmacological uses. In our recent publications, we conducted a comprehensive review of the available evidence about the potential of BP as a great pool of nutrients and bioactive compounds to tackle major aspects of age-related disorders such as neurodegeneration and tumorigenesis [1,2].

Age-related diseases are generally a result of a convoluted interplay of diverse factors, including environmental and lifestyle ones, which drive diverse pathophysiological processes. While these processes are not always well understood, many of them are becoming widely accepted as major hallmarks of aging and as underlying processes, at least partly, in the settlement of age-related diseases such as NDDs, cancers, and metabolic, cardiovascular, musculoskeletal, and other inflammatory diseases. However, despite the colossal effort of the scientific community and the countless research works that are published every year, the conditions and “thresholds” that make aging hallmarks, either separately or jointly, culminate in pathological conditions remain elusive. Epidemiological data revealed that aging hallmarks do not inevitably culminate in what is known as age-related diseases, suggesting that the interplay of modifiable parameters such as lifestyle factors and individual variables such as genetic and epigenetic parameters could play a key role in these diseases [3]. Recently, we have elucidated the unequaled potential of BP as a rich source of nutrients and bioactive compounds that may tackle a wide range of aging-related alterations, in addition to its well-documented antioxidant and anti-inflammatory potentials [1,2]. Therefore, we deemed it necessary to comprehensively explore the compelling prospects of this bee-crafted cocktail against other major aging hallmarks. This publication is the first in a series of forthcoming ones that seek to constitute a milestone in BP-related research and in bioprospection in general. However, more robust data must be provided to ensure the safety of the compounds mentioned in short- and long-term use.

## 2. Bee Pollen and Aging Hallmarks

**Figure 1:**
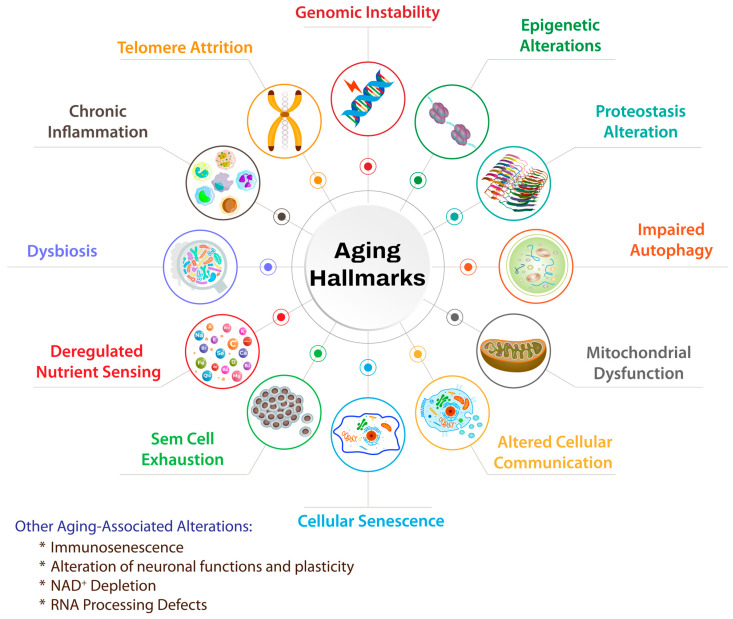
Major aging hallmarks.

Many recent studies tried to categorize and delimit known aging hallmarks. Broadly speaking, the most widely accepted ones within the scientific community include genomic instability, telomere attrition, epigenetic alterations, chronic inflammation, loss of protein homeostasis, impaired autophagy, mitochondrial dysfunction, dysbiosis, deregulated nutrient-sensing, cellular senescence, stem cell exhaustion, and altered intercellular communication [4,5,6] (See Figure 1).

It is important to highlight, however, that some physiological changes, which inevitably accompany the aging process, should also be considered. Immunosenescence, which is a general aspect of aging and is clearly involved in the development and proneness to numerous age-related diseases, is a striking example [7,8]. A similar remark could also be evoked for compromised neural function and plasticity for example. In addition, some authors have recently added other hallmarks such as nicotinamide adenine dinucleotide (NAD^+^) depletion, due to the varied and pivotal roles of this cofactor in nuclear, mitochondrial, and cellular functions [9]. Others have also suggested the defects of RNA processing as a major aging hallmark [5]. Numerous studies that we reported earlier or will report in this work have shown that BP and/or many of its compounds act to ameliorate at least many of these aging hallmarks. We have already reviewed BP’s potential to ameliorate inflammation, oxidative stress, nutrient depletions, gut dysbiosis, autophagy, neuronal functions and plasticity, and immune dysfunctions [1,2,10]. In this section, we will present the available evidence regarding BP’s bioactivities on key aging hallmarks that have a direct well-known interplay with epigenetic regulation, i.e., inflammaging and DNA damage. Thereafter, we will underline some important remarks. It is important to note that inflammaging, DNA damage, and epigenetic alterations are interconnected processes and maintain several bidirectional interplays that culminate in potentiating age-related pathophysiological alterations, as we will see in the upcoming passages.

### 2.1. Chronic Low-Grade Inflammation or Inflammaging

Inflammaging refers to the chronic low-grade inflammation that is settled with aging, and it maintains a bidirectional mutual exacerbation with other diverse aging hallmarks [6]. This chronic inflammation is installed both at the systemic level and in specific anatomical locations and can be evidenced by the increase in pro-inflammatory cells and mediators as well as multiple immune defects, including a weakness in adaptive immunity and an exacerbation of innate immunity [4,11]. Initial pathophysiological mechanisms are, in great part, similar in acute and chronic inflammation, but the subsistence of triggers or the failure of inflammatory response regulation (e.g., the non-resolving of inflammatory response) lead to the persistence of pro-inflammatory signaling for months or years, thus leading to the apparition or exacerbation of inflammation-related diseases [12,13]. It is noteworthy that the latter represents around 60% of all deaths worldwide [13].

Numerous experimental studies have shown that BP, depending mainly on its botanical and geographical origin, may directly tackle a wide range of major chronic inflammation mechanisms. BP has been reported to downregulate pro-inflammatory cytokines (interleukins (ILs) IL-1α, IL-1β, IL-6, and IL-18, tumor necrosis factor alpha (TNF-α), interferon gamma (IFN-γ)), inflammatory enzymes (e.g., inducible nitric oxide synthase, cyclooxygenase 2), inflammation-mediating protein complexes (e.g., NLRP3 inflammasome), inflammatory cell flux and activation, and major inflammatory signaling pathways, i.e., nuclear factor kappa B (NF-κB), mitogen-activated protein kinase (MAPK), and nuclear factor-erythroid 2-related factor 2 (Nrf2) pathways. Furthermore, it has also been reported to upregulate anti-inflammatory cytokines (e.g., IL-10), enzymes (e.g., heme oxygenase 1), and pathways (e.g., Janus kinase 2/signal transducer and activator of transcription 3 (JAK2/STAT3), phosphatidylinositol 3-kinase/protein kinase B (PI3K/AKT)). BP has also been reported to modulate other inflammation response effectors that have other complex networks of biological effects, such as intercellular adhesion molecule 1 (ICAM-1) and transforming growth factor beta (TGF-β). For a detailed review of these bioactivities, see reference [1].

Inflammation resolution is a master phase to suppress inflammatory response chronicity and restore tissular homeostasis, and its failure appears to be a key driver of age-related chronic diseases. Due to these considerations, it is recently becoming largely coveted as a maneuverable target to manage chronic inflammatory diseases [2]. The underlying mechanisms of inflammation resolution remain poorly understood, but it is known that an expanding list of discovered mediators are involved in mediating inflammatory response termination and tissue repair initiation. These mediators are called specialized lipidic pro-resolving mediators (SPMs, mainly lipoxins, resolvins, protectins, maresins, and the recently unveiled docosapentaenoic acid derivatives, which are all derived from omega-3 and omega-6 fatty acids and bind to G protein-coupled receptors to induce cellular effects) [14,15]. In addition to epigenetic mechanisms that appear to be involved in SPM effects, there is also an enhancement in inflammatory cell apoptosis and phagocytosis [14,15]. The latter is mainly ensured through macrophage M2 phenotype-mediated efferocytosis, i.e., apoptotic cell (mainly neutrophils) clearance [14]. Other non-lipidic mediators, such as angiotensin 1−7, IL-10, arginase-1, and annexin A1, and mechanisms such as Treg cell induction [16], are also involved in the complex and insufficiently understood inflammation resolution process but remain less highlighted.

In addition to the effect on the inflammatory process initiation and execution, BP appears to be endowed with an important potential to promote inflammation resolution. Omega-3 and other polyunsaturated fatty acids, the major source of SPMs, are richly present in BP [1]. Targeting SPMs thus appears to hold notable potential in managing the inflammatory basis of many age-related diseases, including cancer, neurodegeneration, and others [17,18]. Heme oxygenase-1 has been shown to plays a key role in inflammation resolution by modulating macrophage polarization [19] and was reported to be significantly downregulated by some BPs [1]. Macrophage polarization is also a recently pursued target in conventional drug as well as in natural product pharmacology [20,21]. Among BP common compounds, kaempferol [22], luteolin [23], quercetin [24], some phenolic acids (e.g., rosmarinic [25] and chlorogenic [26] acids), and proanthocyanidins [22] were reported to regulate macrophage polarization towards the M2 phenotype and suppress the M1 phenotype, while myricetin suppressed microglia M1 polarization in an induced hypoxia environment [27]. Quercetin stimulates macrophage-mediated phagocytosis [28]. Both quercetin and luteolin were found to induce Treg cell differentiation in vivo [29]. Efferocytosis is modified with age and was also recently proposed as an interesting target in tackling inflammaging and related diseases [30]. Resveratrol [31] and some phenolic acids such as protocatechuic acid [32] were reported to promote macrophage-mediated efferocytosis in addition to promoting M2 polarization through different mechanisms. In general, polyphenols, and especially some subclasses such as anthocyanins, are regarded as promoters of inflammation resolution [33,34]. Retinoids (carotenoid metabolites) were also found to promote macrophage M2 polarization and efferocytosis in rodent bone marrow [35]. However, in colon cancer, β-carotene was found to suppress M2 polarization [28]. One should be careful in interpreting experimental results about natural products in macrophage polarization. Indeed, even if it is generally admitted as a beneficial process in inflammation-backed diseases related to aging, macrophage M2 polarization is a rather deleterious event in the tumor microenvironment as it promotes tumorigenesis and tumor growth, while M1 polarization may either encompass pro- or anti-tumorigenic properties in this microenvironment depending on the cellular context [36]. It is important to note that a wide range of BP compounds behave in contradictory ways in malignant and non-malignant cells, as we have reviewed in detail in our recent publication [2]. In addition to vitamin A, vitamins E and K appear to promote efferocytosis [37]. From these preliminary results, we deem it very important to advance experimental research on BP’s potential against inflammaging, and especially, to study BP’s potential against the interplay between chronic low-grade inflammation and other major changes that characterize the aging process and culminate in age-related diseases. This embraces obviously genetic and epigenetic alterations where oxidative and inflammatory events play a crucial role.

### 2.2. Genomic Alterations

Genetic and epigenetic alterations are among the most noticeable other mechanisms that are implicated in the pathophysiology of age-related diseases. Due to the rarity of studies on genome-related bioactivities of BP, we will highlight the available evidence regarding the potential of this cocktail against DNA damage where some consistent experimental data are available. Some elementary data are also available on the potential of some bioactive phytochemicals and micronutrients that are present in BP on telomere attrition. We will review these data at the end of the current subsection.

DNA damage response (DDR) is a complex system of sensors, transductors, and effectors ensuring DNA repair and controlling cell cycle checkpoints to guide DNA replication and cell proliferation and maintain genomic stability and integrity [38]. Cell cycle checkpoints are a cornerstone on which cells rely to prevent the accumulation and propagation of genetic errors during cell division phases [39]. In response to alterations in DNA structure, replication, or assembly, cell cycle checkpoints specifically intervene to arrest or slow down the cell cycle [39]. DDR is a vital process that declines with age, but other factors can compromise it and therefore result in unrepaired or mistakenly repaired DNA damage [40]. The latter is a well-established contributor to ageing by inducing cell death and senescence, but it has also been recently verified to induce inflammation by direct and indirect mechanisms, implicating a newly unveiled role in inflammaging, which is a major culprit in aging and age-related disease [41]. The relation between inflammation and DNA damage is not unidirectional. In fact, DNA damage, the cell senescence that it mediates, and inflammation are mutually coupled to form a deleterious circuit that may culminate in many diseases, including neurodegenerative ones, and that remain tightly linked to altered redox homeostasis and immune response [42].

DNA damage is also an important trigger of epigenetic alterations, which in turn clearly act as inflammation triggers [41]. Moreover, preclinical and clinical evidence revealed that DNA damage may induce a wide range of alterations in mitochondrial structure, dynamics, and function, not only being limited to redox imbalances, but also covering mitochondrial DNA mutations, impaired mitophagy, aberrant metabolic signaling, and other defects [43]. Damages in mitochondrial DNA, which is by nature highly prone to such damages, can also have deleterious effects, which may culminate in transmitted mutations and apoptotic cell death, especially if coupled to alterations in mitochondrial dynamics and mitophagy [44,45]. These mitochondrial alterations may therefore drive aging-related pathophysiological mechanisms and participate in the pathogenesis of many diseases, including NDD, cancer, and related risks such as metabolic and cardiovascular diseases [44,45]. In addition, DNA damage affects nearly all other known aging hallmarks and is thus considered as a key player in the overall aging process [46]. Among small molecules that have been investigated for such applications, polyphenols, mainly flavonoids, and other natural compounds and nutraceuticals occupy a prominent place. A great number of these compounds are widely present in BP, as we will see hereinafter.

An in vitro study reported that an ultrasound-assisted ethanolic extract of *Castanea sativa* BP drastically reduced DNA damage byproducts by 34% [47]. The authors reported that used extract was markedly rich in phenolic compounds (32.18 mg GAE/g), including a particularly high content of rosmarinic acid (5135 mg/kg) and considerable quantities of other phenolics (mainly apigenin, vitexin, pinocembrin, hyperoside, and others), but also contained carotenoids such as β-carotene, β-cryptoxanthin, lutein, and zeaxanthin. Another study of *Actinidia arguta* BP reported that aqueous and ethanolic extracts exhibited a protective role against experimental DNA damages as well as a potent cytoprotective effect of mice lymphocytes against hydrogen peroxide [48]. This study noted that the ultrasound-assisted ethanolic extract was the most active (completely abolished DNA damage at 0.25 mg/mL) and the richest in total phenolic content among all tested extracts. The different extracts under analysis were aqueous and ethanolic, and some were assisted by heat and others by ultrasounds. An in vitro study of an ultrasound-assisted ethanolic extract of a Chilean multifloral BP also found that it was effective in preventing DNA strand breakage [49]. This study compared twelve BPs for their phenolic content and antioxidant activities but only tested the richest sample in phenolics (15.32 mg GAE/g) in a simulated in vitro digestion system. This sample showed a potent preventive effect against DNA damage. The total phenolic content, concentration of cinnamic acid, myricetin and quercetin (which were the major phenolics in BP sample), and bioaccessibility of these compounds were generally higher in digesta samples from intestinal tract than those from gastric or buccal simulated milieux.

In these studies, antioxidant compounds have generally been considered the main actives standing behind DNA damage prevention. This is normal as oxidation is the main mechanism by which environmental and endogenous toxicants and other offenders drive DNA damage, as we have seen. However, we have already elucidated that phenolic compounds are not always the only ones responsible for the antioxidant potential of BP [1]. Despite the examples that we have just enumerated, specific studies of BP as a protector against DNA damage remain very rare. The few ones that have been conducted so far showed very encouraging results, an outcome that is expected as BP is one of the most potent antioxidant and anti-inflammatory cocktails that we know in nature. A guided investigational effort should therefore be directed to bioprospecting for other compounds that we may not know in BP, as well as for very important aspects that remain still almost unstudied, such as synergistic, and obviously the possible antagonistic, effects of diverse bioactive combinations that naturally exist in BP.

Polyphenols are generally supported with strong preclinical evidence as tacklers of DNA damage and other aging-related biological alterations, and thus, they manifest an important potential in countering age-related diseases. Compellingly, these compounds have also been found to promote destructive events in malignant cells such as DNA damage, cell cycle arrest, and redox homeostasis alteration in favor of oxidative stress [50].

Flavonoids seem to be collectively endowed with an inhibitory potential on DNA damages. Many human observational studies have reported that the total intake of flavonoids, or in some cases of specific subfamilies such as anthocyanins or flavanols, is associated with a marked to very marked reduction in the risk of developing many types of cancer [51]. At the molecular level, many ubiquitous flavonoids in BP have been shown to exert inhibitory effects on DNA damage via diverse mechanisms ending up in preventing DNA damage or promoting DNA repair. Among known mechanisms, flavonoids, in addition to preventing oxidative and inflammatory events, may enter in the DNA double-helix and stabilize it, rendering it less vulnerable; bind to the DNA phosphate backbone; groove-bind to DNA bases; interact with chromatin, thus inhibiting diverse proteins, especially many enzymes that regulate genetic signaling; or, at least in the case of malignant cells, act with DNA intercalation [52,53]. Although the mechanisms of DNA damage modulation remain not fully understood, many BP flavonoids are known to act through them. This includes apigenin, catechin, chrysin, epicatechin, hesperidin, kaempferol, luteolin, myricetin, naringenin, quercetin, resveratrol, and some of their derivatives [51,52,53,54,55,56,57]. Quercetin, which is one of the most studied flavonoids, showed a very strong binding potential to DNA, thus resulting in a potent inhibition of DNA amplification and cancer cell proliferation [58]. Bimodal behavior against oxidative mechanisms depending on cellular context and bioactive compound dosage appear to be shared among flavonoids [59]. Kaempferol has also been described for similar activities, i.e., for suppressing DNA damages in healthy cells and inducing them in cancerous cells, with breast cancer cells being the most sensitive [59]. Similar observations were reported for apigenin, luteolin, and quercetin [57,60]. To explain this bifunctionality, some mechanisms have already been suggested. This mainly implies the structure of molecular functional groups and the role of copper ion-mediated chelation of flavonoids and their concentration levels in the DNA microenvironment (higher doses appear to be more protective) [59,61,62], as well as other specific cell-selective properties, as it was reported for luteolin [57].

In addition (or, sometimes, consequently) to their important potential against oxidative and inflammatory mechanisms and roles in cell death and clearance regulation, which we have previously reviewed [2], many phenolic acids have also been endowed with DNA damage prevention qualities. A phenolic acid mixture, containing caffeic, chlorogenic, ferulic, protocatechuic, and vanillic acids, which are all present in BP, and two other phenolic acids (this mixture was prepared as an imitation of a phenolic acid composition of a *Panax ginseng* variant), was recently reported to significantly prevent ultraviolet-induced DNA damage in a concentration-dependent manner in human fibroblasts [63]. Another in vitro study reported that caffeic and syringic acids (both present in BP) tested separately exhibited a protective effect against DNA damage induced by snake venom in a human leukocyte cell line [64]. Gallic acid was found to potently promote DNA damages in some cancer cell types and to be an especially selective and potent tumor-suppressing agent in colorectal cancer, with absent toxicity on tested human lymphocytes [65,66]. As we have seen for flavonoids, many phenolic acids were reported to prevent DNA damages in healthy cells and to promote them in cancer cells. Among the examples that are present in BP, we cite chlorogenic, ellagic, ferulic, and rosmarinic acids, which were reported to have pronounced potentials either as cancer-preventive or as cancer cell-damaging agents [65,66,67,68,69]. Note that caffeic and ferulic acid are among the major metabolites of chlorogenic acid in the body [70]. All of these phenolic acids have been shown to be potently active in the studies that we have cited and are known to be among the major ones in many BP types.

In addition to phenolic compounds, carotenoids are also ubiquitous compounds in BP and may encompass preventive and corrective effects against DNA damage. Antioxidant potential and the preventive role of carotenoids against oxidative stress- and age-related diseases is widely known and reported in the literature [71,72,73]. Carotenoid effects against DNA damage may obviously not emanate only from their known antioxidant potential, but further studies are needed to elucidate this eventuality. Some members of this family act as vitamin A sources in human organisms and are referred to as provitamin A carotenoids, with β-carotene being the main representative, in addition to other precursors such as α-carotene and β-cryptoxanthin [74], which are also found at high levels in some BPs [75]. Other non-provitamin A carotenoids include lycopene, lutein, and zeaxanthin [74], which are also frequent in BP [2]. Many studies reported a preventive effect of DNA damage by combining carotenoids with each other or with other dietary antioxidants such as vitamins C and E and selenium [76]. β-cryptoxanthin was found to protect against experimentally-induced photodamages to plasmid [77] and mitochondrial [78] DNA in vitro and in animal models, respectively. Serum levels of provitamin A carotenoids (α-carotene, β-carotene, and β-cryptoxanthin) were reported to correlate with enhanced DNA repair in humans bearing a specific gene variant of a DNA repair gene in men with prostate cancer treated with finasteride [79]. Lutein was reported to exert an anticancer effect by promoting DNA damages in lung cancer and other cancer cell lines [80]. Lutein and zeaxanthin accumulate in the human retina, where they reduce photodamage through suppressing DNA damage among other mechanisms, such as reducing inflammation and enhancing cell proliferation [71].

Lycopene is a potent antioxidant and one of the most potent singlet oxygen quenchers and free radical scavengers among carotenoids [81]. Lycopene has been shown to mitigate oxidative stress more potently than β-carotene, β-cryptoxanthin, lutein, and zeaxanthin [71]. It has been reported to protect against experimentally induced oxidative DNA damage in diverse cell lines [81,82] and to dramatically reduce DNA fragmentation in a colitis murine model [71]. In prostate cancer cell lines, lycopene was reported to both increase and reduce oxidative DNA damage, while in breast cancer cell lines, it was reported to potentiate the DNA damaging effect of quinacrine [83]. Lycopene is not only a “tomato mark”. BP has also been reported to contain important amounts of this well-studied carotenoid. A recent study reported that lycopene content in fresh and dried tomato was 25.4–33.5 and 701–1181 mg/kg, respectively, depending on the harvest period [84]. Lycopene was found to be present at substantial amounts in some BPs (59.18, 49.67, and 42.55 mg/kg in *Eucalyptus* spp., *Castanea sativa*, and *Erica* spp. BPs, respectively [85]).

Besides the major bioactive phytochemicals of BP, many abundant micronutrients in this matrix, such as vitamins C [86,87], B6 [88,89], B9 [90,91], B12 [92,93], and E [94], and minerals such as zinc [95,96] and selenium [97,98] have been shown to prevent DNA damage in different pathophysiological contexts and with different mechanisms.

In addition, these micronutrients are widely described for their possible other diversified antitumor effects, as is the case for example for selenium [98,99] or vitamins A, C, and E [100,101], as well as for their multiple neurodegeneration-tackling effects [102,103]. Furthermore, some of these nutrients have been reported to intricately modulate DNA damage depending on the cell type, i.e., triggering DNA damage and oxidative events in the studied malignant cells (e.g., vitamins C [86] and E [104] and zinc [95]) and preventing DNA damages in healthy cells (see the references above). Despite these well-founded data, studies of BP’s effect on DDR remain rare and are strongly encouraged. Further preclinical studies are needed, and coordinated efforts must be made to gather further information about BP composition and its determinants around the world.

In addition to tackling the occurrence of DNA damage and its subsequent culminations, BP and its compounds may modulate a key effector that is involved in DDR but have other complex networked physiological and pathological effects, namely poly(adenosine diphosphate-ribose) polymerase 1 (PARP1). The latter is a ubiquitous enzyme intricately involved in a myriad of biological mechanisms, including inflammation, DDR, and cell cycle regulation, as well as in promoting nerve cell death and malignant cell survival [105,106]. In addition to cancer, PARP-1 hyperactivity is implicated in the pathogenesis of many metabolic, cardiovascular, and neurodegenerative diseases and generally in inflammatory and aging-related diseases [105,106]. PARP1 targeting has been successfully used in some cancers and is actively studied in many age-related diseases, including cancers [107] and NDD [108]. As many natural compounds have shown important modulatory effects on PARP-1 but have not been clinically tested, neither in cancer nor in neurodegeneration, it is important to remember that cell death is the ultimate desirable goal of anticancer therapies, while it is the ultimate fatal condition to avoid in neurodegeneration. Indeed, mechanistic interventions that may be wanted from targeting PARP-1 will be significantly different between the two pathologies. PARP-1-based prospection in neurodegeneration must especially consider the potential toxic effects of such approach, mainly emanating from activating PARP downstream signaling pathways, which are involved in a complex network of physiological and pathophysiological events.

Experimental inhibition or genetic knockdown of PARP-1 has been shown to reduce Aβ42 isoform plaques and consequently reestablish locomotor activity in transgenic *Drosophila* models of AD; in addition to a great increase in nicotinamide adenine dinucleotide (NAD^+^, of which depletion is a well-known and important hallmark of neurodegeneration) levels in the brain of studied flies; a strong decrease in DNA transposable elements, which are known to be excessively transcribed in many NDDs, including AD and PD; and an amelioration of life expectancy [109]. This study in the most used model of AD shows that PARP-1 inhibition could be a novel path in the ardent journey to search for preventive and therapeutic means for neurodegeneration.

To the best of our knowledge, there is no published study about the effect of BP as a whole product on PARP-1. However, there is a growing number of studies on BP compounds. Many BP phenolics have been shown to inhibit PARP-1. Among the most ubiquitous ones in BP, apigenin, delphinidin, luteolin, myricetin, many catechins, naringenin, quercetin, resveratrol, and hesperetin; and glycosylated derivatives of some of these molecules, namely hesperidin, isoquercetin, and naringin, were all found to exert inhibitory effects on PARP-1. All of these compounds were reviewed in [106]. Kaempferol was also found to potently exert such an inhibition [110]. Isorhamnetin was found to induce cleavage of PARP proteins [111]. Many phenolics have shown potent activities that may exceed that of synthetic clinically adopted PARP inhibitors, but it is noteworthy that glycosylated forms are generally less potent but may display high selectivity to some cells (e.g., isoquercetin, naringin, and hesperidin to breast cancer cells) or a safer profile (e.g., isoquercetin compared with quercetin) [106]. That is even more important, as major BP flavonoids are known to be ubiquitously present in glycosylated forms in BP.

Nicotinamide, the water-soluble form of vitamin B3 and known precursor of NAD^+^, is present in high quantities in BP [112]. Considering that a nicotinamide-like moiety characterizes most of the known PARP-1 inhibitors (we have seen some exceptions, such as the flavones apigenin and luteolin) [106], nicotinamide itself was found to possess a marked PARP-1 inhibitory potential. It is involved in numerous vital metabolic roles, including those related to neuroprotection and anticancer mechanisms, such as enhancing mitochondrial function and dynamics; tackling oxidative stress; suppressing numerous pro-inflammatory processes, including neurological ones; and regulating autophagy [113]. By a feedback mechanism, nicotinamide inhibits PARP-1 and, interestingly, SIRT1, another key effector in DNA repair, carcinogenesis, aging, and cell death; the two effects thus result in the suppression of NAD^+^ depletion [114].

Telomere attrition is another genomic alteration that is well established as a factor of aging and aging-related pathophysiological events. Telomeres are built with TTAGGG hexanucleotide repeats bound by a set of telomere-capping proteins that protect telomeres from DDR and ensure the regulation of their functions [115]. Telomere attrition occurs as the ability of cells to fully replicate DNA ends declines with age. Such a decline is driven by normal aging process (number of successive cell divisions) but also by diverse internal and external factors that exacerbate it [116]. When a critical telomere length is reached, the latter become vulnerable to DDR by being unable to sufficiently bind capping proteins. This situation induces a cell proliferation arrest and permanent foci of DDR with a resistance to DNA repair mechanisms, in addition to other aging-related events such as stem cell alterations and sustained pro-inflammatory processes [115]. Differently to what is observed in in vitro assays, telomerase, the enzyme that is the main responsible for telomere extension and maintaining, cannot assemble alone and needs other proteins for its assembly and activity in human cells [117]. Diverse studies have suggested that foods that are generally rich in antioxidant and anti-inflammatory compounds appear to tackle telomere shortening [118]. Polyphenols are reported to preserve telomere length in healthy cells and to promote telomere attrition and cell death in malignant cells [119]. Some frequent BP compounds such as quercetin were found to tackle telomere shortening and cellular senescence [120]. Higher intake of carotenoids have also been reported by observational studies to preserve telomere length and integrity in humans [119]. Many nutrients that are present in BP, such as dietary fibers [119], unsaturated fats (e.g., omega-3 and monounsaturated fatty acids [121]), vitamins (e.g., vitamin C [87], folic acid [122], and alpha-tocopherol [123]), and minerals (e.g., selenium and zinc [124]), have been reported to promote diverse mechanisms that prevent telomere attrition and preserve telomere integrity.

Interactions among different BP compounds in such effects may be of great importance and are still not studied. Resveratrol for example, which is also present in some BPs, have been largely reported to suppress telomere attrition and cellular senescence [125]. This stilbene, when associated with copper, produced a pro-oxidant effect but still induced a series of anti-aging effects, including the reduction of cellular senescence and telomere attrition in rodents [126].

**Figure 2:**
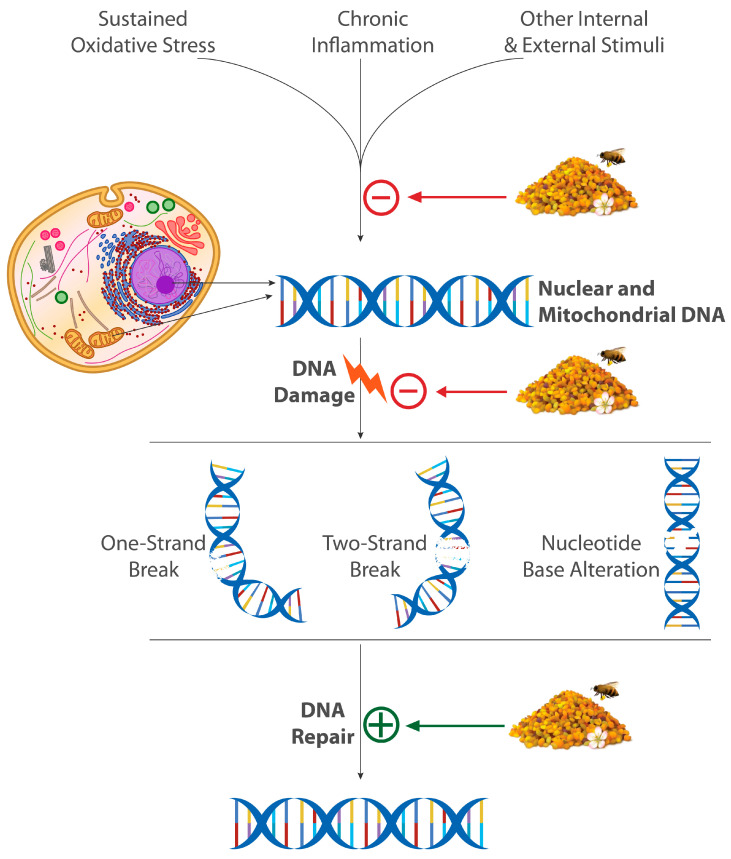
Potential targets of BP and its compounds in DNA damage and repair.

In summary, BP’s potential to modulate DNA damage and repair mechanisms could be broadly resumed in three major mechanisms, namely the antagonism of DNA damage-generating stimuli, in the DNA damage process, and in DNA repair mechanisms. A general presentation of this potential is presented in Figure 2.

### 2.3. Important Remarks

In different subsections of this paper and in our other recent publications [1,2,10], we have seen that a wide range of BP compounds tackle diverse events that are directly and indirectly involved in DNA damage and other genomic and epigenomic alterations, as well as in cellular senescence. Bioprospection in BP, and in natural products in general, must focus on deciphering bioactive compounds and synergies to prevent DNA damages, promote DNA repair mechanisms, suppress other genomic vulnerabilities such as telomere alteration and epigenetic aggressions, and complement theses bioactivities through the hindering of other pathophysiological mechanisms that maintain bidirectional exacerbation loops with genomic and epigenomic alterations. In this context, some great challenges must still be overcome. We think that the most important ones at our current level of knowledge are the problems of reduced bioavailability of natural products in general and phenolic compounds in particular, as well as the poor understanding of both pharmacodynamics and pharmacokinetics of natural antioxidants and the exact regulation of DDR in the different contexts of healthy, at-risk, or damaged (e.g., malignant, senescent, or degeneration-condemned) cells. The potential of some polyphenols and other molecules that we have seen to intricately modulate oxidative events, DNA damage, and cell death mechanisms by promoting them in malignant cells and mitigating them in healthy cells should deeply draw the attention of scientists. This urge to figure out the implicated mechanisms and use acquired knowledge about these properties in conceiving novel therapeutic means or boosting the existing ones, as well as to tackle drug toxicity, still constitutes one of the most dreadful impediments in managing complex diseases. Understanding when this modulatory potential is beneficial and when it may become deleterious is a crucial need, firstly to avoid unknown hazards that may be linked to these products if isolated and used at high doses, and secondly to take advantage of their interesting and pleiotropic bioactivities. The distinction between these two axes of activities obviously appears to be very hard to delineate, but experimental evidence and data from observational and from some clinical studies focusing on polyphenols also appear to be very encouraging. A new era of rigorous and objective, but open-minded and holistic, research projects is needed more than ever as we are facing an endless pool for bioprospection and a set of very devastating diseases that remain ununderstood, untreatable, and unpreventable.

It is important to keep in mind that the interplay of aging hallmarks is still not clearly elucidated and that auto-sustained loops exist between these hallmarks. Aging process and age-related diseases generally occur as specific sets of these hallmarks and their interactions. On the other hand, BP is a great multitargeting arsenal that is rich in numerous compounds that tackle nearly all known systemic aging hallmarks, as well as localized aging phenomena such as neurodegeneration. The challenge is therefore to gather the available substantial amount of elementary preclinical data and translate it into meaningful knowledge for clinical practice and real-world interventions.

## 3. Bee Pollen Compounds and Epigenetics

The evidence for BP potential in epigenetic modulation is also consistent and very noteworthy. Although the studies of BP as a whole product are still quasi-absent, we will detail a large body of evidence to unveil such potential. To narrow the spectrum of examples due to the large diversity of age-related diseases and their underlying mechanisms, we will focus our analysis in this section on neurodegeneration and cancer. We made this choice because these are the most challenging burdens of worldwide healthcare due to the great insufficiency in their understanding and the scarcity in their management arsenals, which unfortunately culminate in a lack of efficient preventive and curative means, especially in neurodegeneration.

### 3.1. Epigenetic Regulation and Age-Related Diseases

Epigenetic regulation, being mediated by three major types of mechanisms, i.e., DNA methylation, histone modification, and non-coding RNA (ncRNA), is a major genome modulator that may shape the human phenotype, and, thus, deeply contributes to defining health and disease factors and critically determining numerous pathological events, including those implicated in age-related diseases [127,128]. Although playing a key role in genetic expression and being inheritable and transmissible during cell division, epigenetic modifications can be reversed and are fortunately “reprogrammable” or “erasable” due to pharmacological and nutritional interventions [127,128,129]. In this context, BP, as an unequaled nutrient resource and a rich pool in bioactive compounds, may represent a potential tool to carry out such interventions. Before developing BP’s potential, we will briefly summarize the three major epigenetic mechanisms:

1. DNA methylation is the fixation of a methyl group predominantly to CpG (cytosine-guanine in the 5′-3′ direction) dinucleotides by DNA methyltransferases (DNMTs), thus forming 5-methylcytosine (5mC) [130]. The methyl group originates mainly from S-adenosylmethionine (SAM), which acts as a universal methyl donor to DNMTs [131,132]. SAM originates from the methionine cycle, which in turn is known to be a part of the larger one-carbon metabolism (OCM) network of metabolic pathways that involve many micronutrients as methyl donors to produce SAM (we will see them below) [131]. The DNA methylation profile appear to be a reliable indicator of epigenetic age in diverse organs and functions and is being used to define many age predictors that become commonly known as epigenetic clocks [133]. These clocks are subject to extensive research in aging and present a highly accurate mean of expressing chronological age and evaluating its distinction from biological age according to DNA methylation level discrepancies between normal and pathological aging [133,134], a gap that is significantly big in some age-related diseases such as neurodegeneration [135].
2. Histone post-translational modifications include acetylation, methylation, phosphorylation, and ubiquitination. These modifications, which may alter chromatin structure by steric hindrance or induce physicochemical modifications of histones, may happen separately or in combination and therefore result in an endless number of combinations and consequent biological responses [127]. In all cases, these modifications will act as marks and will trigger the recruitment of chromatin-modeling complexes, which are proteins that may be called “writers” (grab these marks, e.g., histone acetyltransferases (HATs)), “readers” (read them), or “erasers” (delete them, e.g., histone deacetylases (HDACs)), depending on their function [127].
3. ncRNAs: Advances in genetic detection and isolation techniques and the achievement of the Human Genome Project strikingly permitted us to know that only 1–2% of the human genome codes for protein and that ncRNAs are a very functional and regulatory network involved in controlling all biological processes and playing crucial roles in the pathophysiology of diverse human diseases [136,137]. These RNA transcripts are roughly classified in “housekeeping” and “regulatory” ncRNAs, while the latter are simply distinguished according to their sequence length into short and long ncRNAs and are emerging as promising biomarkers and therapeutic targets in numerous diseases, including NDDs and cancers [136,137]. Among short ncRNAs, microRNAs (miRNAs) have been extensively studied for their post-transcriptional regulatory role (messenger RNA silencing) of gene expression and are widespread epigenetic regulators, which are present in diverse cell compartments, including cytoplasm, mitochondria, intracellular vesicles, and others [138]. Extracellular vesicles, which are known to play critical roles in variety of cellular communication and pathophysiological processes, also contain ncRNAs in their cargos and may thus play important roles in regulating gene expression through regulatory ncRNAs such as miRNAs [139]. We will see that BP compounds may affect all epigenetic regulators that we have briefly described, including miRNAs, and that extracellular vesicles may present an enormously rich and complex BP component that is still completely untapped.

To illustrate the importance of epigenetic alteration in age-related pathology, we will highlight major mechanisms in two major disease examples, namely carcinogenesis and neurodegeneration.

In addition to genetic alterations that we have briefly described, epigenetic mechanisms are other major actors in the complex interplay, leading to the carcinogenesis process and involving environmental and body-specific modulable factors. In cancer pathogenesis, it is very important to note that epigenetic information is settled since the very early phases in life (beginning just after fecundation), and this may be of extreme importance as epigenetic alterations and carcinogenic processes can affect pluripotent cells, either stem or embryonic, thus lastingly influencing cellular and organism fate [140]. Major epigenetic mechanisms in carcinogenesis may be summarized into three main groups. First, DNA methylation and demethylation levels and distributions are altered in diverse tumors, resulting in the hypomethylation in large areas of chromosomic material and consequently in a more abundant and vulnerable euchromatin, genomic instability, and overexpression of diverse oncogenic genes, or inversely in hypermethylation in a few regions, which may imply silencing crucial genes such as tumor suppressors, which is a common cancer hallmark and is one of the most studied and widespread orchestrators of tumor genesis and metastasis [141,142]. Second, histone post-translational modifications and/or ATP-dependent remodeling can modulate histone conformation and consequently the accessibility of transcription factors and other mediators and/or induce gene expression aberrances that can result in promoting pro-carcinogenic gene expression, especially those related to migration, invasion, and metastasis [140,141,142]. Third, alterations in non-coding RNA expression may drive alterations in chromatin dynamics and conformation; diverse interactions in the nucleus and cytoplasm; and increased or decreased binding to various target genes, and thus, they may promote gene expressions that are involved in initiating or developing tumor formation [140,141].

Regarding neurodegeneration, DNA methylation has been proven to be crucial for neurogenesis, neurodevelopment, and numerous neurological functions, including memory and cognition and diseases including NDDs, although the exact underlying mechanisms are not always clear [143]. However, it is still not understood whether DNA methylation itself is a driver of the ageing process or just a mediator trained by other molecular and cellular mechanisms [133]. Altered DNA methylation is a common aspect in major NDDs, including Alzheimer’s (AD) and Parkinson’s (PD) diseases. Interestingly, these diseases manifest a similar pattern of epigenetic alterations in a significant set of CpG sites [144], supporting the possibility of common NDD pathogenesis that differentiate thereafter according to unknown or partially known interacting factors.

Alterations in the regulation of histone acetylation are tightly linked to aberrant protein deposition and impaired neuronal homeostasis and plasticity [145] and therefore may play a crucial role in neurodegeneration pathophysiology. Overexpression of some HDACs and impaired acetylation in general is associated with pathogenic Aβ, tau, α-synuclein, and huntingtin aggregation [145,146]. The inhibition of HDACs and upregulation of HAT-mediated acetylation have been reported to reduce aberrant protein deposition and other neurodegeneration traits in animal models and were proposed as potential therapeutic targets to restore gene transcription and correct cognitive decline and other neurodegenerative processes [147,148]. However, before engaging in such an appealing perspective, a pivotal question must first be answered: are these acetylation aberrances a trigger or a result of neurodegeneration? Obviously, both mechanisms may exist, but conclusive evidence is still lacking. Altered histone acetylation is also strongly involved in neuroinflammation [149,150] and in a myriad of other neurodegeneration-triggering mechanisms [151,152]. Histone and chromatin modifications, as well as ncRNA actions, are also implicated in age-related neuronal function and survival [153]. Moreover, histone modifications and chromatin remodeling were linked to nerve regeneration in humans, for example, through restoring myelination by oligodendrocytes [154]. Likewise, histone modifications are very tightly linked to the pathophysiological mechanisms in diverse types of cancers and are successfully investigated, and sometimes clinically used, as therapeutic targets (for recent reviews, see [155,156]).

Wingless-type mouse mammary tumor virus integration site (Wnt)/β-catenin is a crucial and extensively studied signaling pathway involved in a myriad of biological processes related to cell differentiation, renewal, proliferation, and fate determination through the whole lifespan, and thus, it has a great and multifaceted impact in many pathophysiological events, particularly those related to cell alterations such as cancers and neurodegeneration [157,158]. The Wnt/β-catenin pathway is substantially regulated by epigenetic mechanisms involving numerous genes and signals and implying all epigenetic ways, i.e., DNA methylation, histone modification, and microRNAs [159,160]. β-catenin is particularly implicated in DDR by targeting many of its genes [161] and affects many cell cycle regulators but also many metabolic signaling pathways, making its abnormal levels, which may result from different mutations in the Wnt canonical pathway, deeply involved in carcinogenesis, metastasis, and resistance to chemotherapy [157]. Wnt/β-catenin alteration is also markedly involved in altering microglia, astrocyte, and oligodendrocyte functions; impairing neuronal survival and regeneration; affecting synaptic plasticity and transmission; and promoting aberrant protein deposition and neuroinflammation, among other effects [158].

### 3.2. BP as a Valuable Source of Epigenetic Modulators

Many BP ubiquitous phytochemicals have been found to modulate major epigenetic mechanisms. Some of them have potently suppressed oncogenic epigenetic signaling and promoted the epigenetic induction of tumor suppressor gene expression in experimental studies. Polyphenols are widely reported for their countless effects resulting from epigenetic modulatory mechanisms in neurodegeneration and cancer pathophysiology (good recent reviews can be found in [162,163,164,165,166]). All of these effects are not limited to differentiated fully functional cells. The three major mechanisms of epigenetic modulations have also been verified for numerous polyphenols in cancer stem cells, which play a crucial role in cancer renewal and resistance (reviewed in [163]). Indeed, we will hereinafter give only some of the most relevant examples of BP ubiquitous compounds.

Kaempferol inhibition of DNMT1 is for example an interesting mechanism which results in an increase in the demethylation of disheveled-associated antagonist of β-catenin 2 (DACT2), i.e., its reactivation, which has been shown to suppress colorectal cancer cell proliferation and migration [162]. DACT2 is known to be depleted in many cancers, and its decreased levels closely correlate with increased occurrence, development, invasion, metastasis, and overall poor prognosis in many cancers [167]. In silico and in vivo studies have reported that kaempferol was an HDAC inhibitor against all tested deacetylases [168]. Marked inhibitory activity on HDACs was especially confirmed in human cell lines of hepatoma and colon cancer and correlated with the reduced viability and proliferation of these cells [166,169]. In addition, this flavanol upregulated the expression of miR-340, which is an apoptosis inducer and cell proliferation inhibitor [168]. Interestingly, kaempferol has also been shown to mitigate the Warburg effect in human colon cancer cell lines by inhibiting aerobic glycolysis through the upregulation of miR-339-5p (a tumor suppressor that is altered in some cancers), an effect that was further enhanced when associating kaempferol with a miR-339-5p mimic [170].

Quercetin inhibits DNMTs, HATs, and HDACs, with consequent activities, including histone acetylation enhancement and an increase in DNA demethylation in the promoter regions of apoptotic genes, thus resulting in the upregulated transcription of proapoptotic mediators in many cancer experimental studies [162,171]. In breast cancer, quercetin enhances the epigenetically modulated expression of breast cancer genes (BCRA 1 and 2), an effect where HAT-mediated regulation of β-catenin appears to be involved [172]. It was also found to be very effective in reversing epigenetic silencing of androgen receptor in prostate cancer and in potentiating adopted drugs for such effects [169]. In a wide range of human cancer cell lines, quercetin has also been reported to upregulate some anticarcinogenic, anti-proliferative, and proapoptotic miRNAs; downregulate some oncogenic and metastasis-mediating (promoting migration and invasion) miRNAs; and to correct aberrances of other antiproliferative, anti-angiogenic, and proapoptotic ncRNAs (these effects were recently reviewed in [169,173]), in addition to obviously exert a myriad of anti-proliferative, anti-metastatic, anti-angiogenic, proapoptotic, and chemo-sensitizing effects [174,175]. Quercetin was also reported to activate SIRT1 (which is also a histone deacetylase appearing to selectively prevent aberrant methylation [176]) in some cancers [162] and its AMPK and mTOR downstream pathways, with histone modification and DNMT regulation likely being involved in these effects [177].

Resveratrol has been reported to reverse epigenetic alterations through miRNAs that are involved in ovarian cancer, inflammatory processes, NDDs, and other diseases and cells [178,179]. Resveratrol is especially known to upregulate a vast array of proapoptotic and tumor suppressor miRNAs or corresponding genes that are commonly silenced in diverse cancers [142]. It also corrects the altered expression of many long ncRNAs involved in carcinogenesis initiation and progression and malignant cell proliferation and apoptosis in different cancer cell lines (implications in cancer are reviewed in [173]). Resveratrol also modulates HATs and HDAC and interestingly regulates other chromatin proteins than histones [179]. Accordingly, resveratrol inhibited the epigenetic reader metastasis-associated protein 1 and its associated cell signaling, tumor progression, and metastasis in prostate cancer [180]. Furthermore, importantly, this phytochemical was found to prevent epigenetic mark transmission from rodent mothers to offspring. This valuable quality was verified in neurodegeneration and some metabolic disorders and manifested in DNA methylation, histone modification, and miRNA signatures, thus unveiling an important trans-generation preventive role [179,181]. In addition, trans-resveratrol administration to mother rats, even at low doses, upregulated the most abundant miRNA in the brain, viz., miRNA-124, and resulted in the enhancement of neuroprotection, neurodevelopment, and neuroplasticity since early life [182]. Resveratrol is also widely known to upregulate SIRT1 [162,179,183], an effect which appears to markedly stand behind a large part of its roles in many inflammatory, oncogenic, and neurodegenerative processes [179,184,185]. Epigenetic regulation by resveratrol is also involved in its modulatory effects on many vital signaling pathways such as AMPK (through SIRT1 upregulation and via other indirect mechanisms), mTOR, and insulin/insulin-like growth factor-1 [179,183].

Apigenin has especially been characterized by its marked inhibitory potential on HDACs in diverse cancers [168,169]. This multipotent flavone was also found to greatly induce, through epigenetic modulation, activating transcription factor 3 [169], which is a major regulator of immune response and many metabolic and other biological processes, notably manifesting in its crucial protective roles against many cancers [186]. Apigenin upregulated some proapoptotic miRNAs and downregulated oncogenic ones in human cell lines [187,188,189]. Myricetin is another BP and plant ubiquitous flavone that potently inhibits DNMT and upregulates HDACs, especially SIRT1 [169]. Still among flavones, marked DNMT inhibition has been identified as a participant in the strong anticancer effects that have been widely reported for luteolin by many experimental studies [169]. In addition, studies in diverse cancer cell lines have reported that luteolin upregulates a large number of tumor-suppressing and proapoptotic miRNAs and downregulates many oncogenic and anti-apoptotic ones [190].

A series of experimental, mainly in silico and in vitro studies, have unveiled that hesperidin and naringenin drove, through epigenetic modulation in breast cancer, numerous anticancer biological responses, such as increased proapoptotic gene expression, oncogenic gene suppression, and many signaling pathway enhancements, especially in Wnt/β-catenin and its downstream pathways [191]. Naringenin was found to exert neuroprotective and anticancer effects by downregulating the expression of some pleiotropic miRNAs such as miR-25 and miR-17, which are involved in oxidative, inflammatory, and diverse other biological processes, and upregulating the immunomodulatory miR-223 and tumor suppressor miRNA let-7a [169]. Some evidence has also suggested that naringenin possess HDAC inhibitory activity [168].

Epicatechin, epicatechin gallate, gallic acid, quercetin, kaempferol, and resveratrol inhibited DNMT in different cancer types [162,166]. Inhibiting DNMTs and decreasing their gene expression and translation appear to be shared among catechins, including those present in BP, i.e., catechin and epicatechin [192]. Epicatechin, epicatechin gallate, and gallic acid act as inhibitors of HATs [162]. Gallic acid inhibits the majority of HDACs [162], and was found to modulate miRNAs and long ncRNAs, with a special effect on miRNAs involved in DNA repair, while spermidine was reported to regulate chromatin condensation and DNA conformation [193]. Delphinidin, reported as a main anthocyanidin in some BPs [194,195], was found to exert DNMT and HDAC inhibitory activities and to suppress some pro-carcinogenic and metastasis-promoting miRNAs [168].

Genistein, the soybean “mark”, is also the major isoflavone present in BP [196]. Studies in human breast cancer cell lines and animal models have reported that genistein inhibits DNMT1; reduces the promoter methylation of BRCA genes and other tumor suppressors, thus reactivating them and promoting their protective role; enhances chromatin acetylation marks; and modulates miRNAs (e.g., upregulates tumor-suppressive and proapoptotic miRNAs and downregulates the oncogenic ones) in diverse cancer types [172,173,174]. Genistein was also found to promote histone acetylation at starter sites of tumor suppression genes in human cell lines of many cancer types [142]. This phytocompound also corrected the epigenetic loss of the anti-aging protein Klotho in mouse fibrotic kidney. Underlying mechanisms included simultaneous inhibition of histone 3 deacetylation of the Klotho promoter and correction of the promoter DNA hypermethylation by suppressing DNMTs [197]. To complete its wide array of anticancer effects, this phytochemical plays important roles in promoting cancer cell apoptosis and suppressing proliferation, angiogenesis, and metastasis in some cancers through long ncRNAs modulation [173].

Chronic inflammation is a main interacting culprit with epigenetic mechanisms and is a major pathogenic factor in cancer and neurodegeneration, as we have explained. Interestingly, polyphenols may reduce inflammation directly and through epigenetic modulation mechanisms, which will result in mitigating low-grade chronic inflammatory diseases [198] such as cancer and NDDs. A series of experimental studies have verified the significant modulatory potential of polyphenols toward chronic inflammatory response via all known major epigenetic mechanisms. Among those present in BPs, this includes apigenin, epicatechin, gallic acid, luteolin, resveratrol, and other polyphenolic mixes isolated from vegetal sources (reviewed in [199]). The other inflammation-related chronic deleterious phenomenon in cancer and neurodegeneration is undeniably oxidative stress. Epigenetic mechanisms are acknowledged as the mediator between oxidative stress and genetic structures and functions [200]. Oxidative stress may directly damage DNA and chromatin structures or indirectly affect them through inhibiting DNMT binding, affecting histone post-translational modifications, deregulating transcription factors, impairing the genesis and effects of miRNAs, and altering diverse metabolites that are essential for epigenetic mechanisms and implicated enzymes [200,201]. Phenolic compounds are widely reported to modulate this stress through epigenetic regulation. Among many studied phenolics, some of those present in BP, such as apigenin, delphinidin, luteolin, and resveratrol, reduce the Nrf2 methylation by inhibiting DNMTs, mediating its demethylation, and thus promoting reduced methylation of the Nrf2 gene promotor and contributing to the correction of altered levels of this key transcription factor in inflammatory and oxidative regulation [202]. More generally, many phenolic compounds, including some of those present in BPs, have been shown to exert a part of their antioxidant effects through the epigenetic modulation of DNA methylation, histone, and other chromatin protein modifications and/or ncRNA regulation, in addition to obviously inducing miscellaneous epigenetic regulations as a result of their direct antioxidant mechanisms, either inside the mitochondrion or in other cell compartments [164,179,192,200,203].

Numerous effects of phenolic compounds, including some of those present in BP, on epigenetic processes have also been shown to result in neurodegeneration-countering outcomes. This included, for example, ellagic acid, epicatechins, gallic acid, kaempferol, quercetin, and resveratrol [166,179,204,205]. The number of studies investigating epigenetic regulation by phenolic compounds in neurodegeneration prevention and treatment remains far less than studies focusing on cancer. This appears to be mainly due to the poorer understanding of neurodegeneration mechanisms and the scarcity of interventional tools in managing NDDs in comparison with cancers. Small molecules acting as epigenetic modifiers, such as the examples that we have seen, have an important advantage if they can cross the BBB. This will permit, especially at early disease stages, the mitigation of neurodegenerative mechanisms that are known to be epigenetically induced or modulated (e.g., Aβ production, tau phosphorylation and accumulation), a possibility which has been observed with HDAC inhibitors in animal models of NDDs [130].

Another point to consider is that the same epigenetic mechanisms or signals are frequently involved in the pathophysiological issues of both types of diseases. The clearest example could be miRNAs, which usually play a large plethora of physiological and pathological roles. miR-134 is known to be a brain-enriched miRNA that contributes in regulating neurogenesis, neurodevelopment, synaptic plasticity, and neuronal excitability, and it appears to be implicated in many neurological disorders, including AD and epilepsy, where it has emerged as a potential disease-modifying target [206,207,208,209]. This miRNA is paradoxically known to be an important tumor suppressor in many cancers [210,211] and has been markedly downregulated by resveratrol in rat models of AD [212]. Another example without a contradictory appearance is miR-7. This miRNA is well studied in cancer and is known to play a crucial stabilizing role in a number of networked signaling pathways, mediate a series of transcriptional feedback loops, and especially act as tumor suppressor while being silenced by DNA methylation in cancer cells [213]. This miRNA is potently upregulated by quercetin (it was found to be the most potent activator of miR-7 using a nano-scanning investigation) [214]. This upregulation has been found to be particularly useful in suppressing α-synuclein deposition [214]. Experimentally induced overexpression of miR-7 was found to inhibit α-synuclein fibrillation, mitigate neuroinflammation, and protect dopaminergic neurons in animal models [215]. Notwithstanding their complexity and the insufficient understanding of their implications, these shared mechanisms may endow multitarget epigenetic modifiers with a great potential in fighting aging-related diseases, among which cancer and neurodegeneration occupy the foremost place. This is even more interesting in the case of natural safe resources that may be used as nutraceutical and pharmacological tools in humans, such as BP.

The plethora of epigenetic regulatory mechanisms that we have seen for these major examples is clearly important and very promising. One of the most important challenges to resolve in the case of BP as a wholly usable product in human nutrition and medicine is to study the combinations of these bioactive compounds and how they behave when administered together. BP generally contains more than one of these phenolics and other epigenetic-regulating compounds and nutrients that we will see briefly. Studies of synergistic effects remain very scarce. However, the few ones that have been conducted so far reported expectedly encouraging results (see sample reviews in [216,217] for combinations including the example molecules that we have just discussed). In addition to this type of research, many other studies that we do not report here have reported advantageous results when combining the epigenetic regulators that we have seen, viz., phenolics with chemotherapeutic drugs. As we have already discussed, the BP pool around the world remains untapped for the most part. The few BPs that have been characterized in detail from different regions of the world have unveiled a wide spectrum of important epigenetic modifiers, including bioactive phytochemicals and micronutrients, which are frequently present at substantial amounts in this natural and rich matrix. Epigenetic alterations are widely acknowledged among the scientific community to be a main contributor in the pathogenesis of cancers and NDDs, a contribution that generally evolves over many years to settle the confirmed disease state. The presence of such a large spectrum of bioactive compounds in BP may represent a novel and versatile arsenal to lastingly reduce the incidence and accumulation of epigenetic alterations, at least in at-risk individuals. In addition, BP prospection for novel phytopharmaceuticals with potential application in epigenetics should advance at greater pace due to the amount of available evidence and the urgency of epigenetically driven diseases. Complementarily, the available literature has also reported a substantial amount of evidence unveiling the potential of phenolic compounds in regulating numerous aspects of metabolic and cardiovascular disorders (not reviewed in the current work due to the great length of our discussed topic), which are usually approached as culprits in the long-term triggering of neurodegeneration and carcinogenesis. The same thing is widely verified for diverse micronutrients of BP. A problem that remains to be solved with this bee cocktail is the difficulty of using it to selectively target an epigenetic regulator or process. However, due to the networked and complex implication of every known epigenetic process, the great complexity and multifactorial nature of related diseases such as neurodegeneration and cancer, and the complementary activities of BP in many pathophysiological situations (as we are seeing in this work), the molecular approach of preventing and managing diseases appears to have many things to reconsider.

Another big issue for translational research on phenolic compounds in epigenetics is the fact that experimental research works almost always study specific compounds prepared in lab extracts that may differ from the real natural context of bioactive compounds, or, in case of single molecules, evaluate them in an experimental media while overlooking diverse interactions that may occur in complex biological contexts; thus, the analysis incompletely, or even wrongly, understands the underlying mechanisms. In addition, investigations always focus on limited number of epigenetic variables and/or signaling pathways, a reality that alters the credibility of experimental results given that such variables and pathways are known to generally interact with each other and to also be modulated by other mechanisms, either known and missed or unknown, in very complex, intricate, and experimentally hard to reproduce ways. To help to avoid such hindrances, especially when facing the great diversity and complexity of epigenetic mechanisms and phenolic compounds, profitable use must be made of the available online databases that gather a lot of data about molecular compounds, biological matrixes, miRNAs, and other translational tools. High-throughput untargeted technics, involving network pharmacological studies and omics sciences and using new machine learning advances, and in silico studies may save a lot of time and effort and accelerate bioprospection, especially in very diversified and complex resources such as BP. Furthermore, researchers and public and scientific authorities may have the “moral duty” to enrich such databases and settle new ones to make results accessible to other researchers and achieve reliable and sustainable results. In this context, gathering epigenetic marks and adopting reliable profiles of such marks to detect vulnerable pathophysiological contexts to neurodegeneration or cancer early on is an urgent need and will be an unequaled achievement of worldwide coordinated research works. It also goes without saying that analytical and other logistical tools must be accordingly rendered more affordable and accessible to researchers.

BP’s nutritional value is one of its most valuable endowments. Accordingly, the potential roles of nutritional interventions in modulating epigenetic alterations, especially in preventing long-term deleterious “programming” of biological processes and cell fate determination, are solidly based on a large and growing body of evidence. For recent reviews related to aging, neurodegeneration, and cancer, see [129,218,219,220,221,222,223]. Therefore, BP may virtually be a great source of epigenetic modulatory nutrients as it contains almost all known essential micronutrients, generally in marked concentrations, as we have seen. We will briefly describe the evidence-based data of vitamins, minerals, and other nutrients that are frequent in BP, epigenetic programming, as well as consequent prospects for intervention in neurodegeneration and carcinogenesis pathologies.

Vitamin C may be the most important epigenetic-regulating vitamin in BP; notwithstanding vitamin D, which is a widely acknowledged epigenetic modulator [224,225], but very rare studies have reported its presence in BP, as we have seen. One of the seemingly most relevant roles of vitamin C, which was relatively newly discovered, in epigenetic processes is its role as a cofactor of ten-eleven translocation (TET) and jumonji C-domain-containing histone demethylases (JHDMs) [226,227]. TETs are a family of enzymes involved in inducing DNA demethylation by oxidizing 5mC, and they are consequently implicated in numerous biological and pathophysiological processes [228]. JHDMs are a large family of enzymes (33 identified in humans) that are involved in regulating histone methylation and in orchestrating the crosstalk between cancer and inflammation. This involvement has been recently identified as one of the most important mechanisms in tumor occurrence and progression [229]. Vitamin C is a pleiotropic inducer of DNA demethylation (thus inducing demethylation in ~2000 genes in embryonic stem cells for example), while its depletion was reported to result in a quasi-complete suppression of histone demethylation in vitro [226]. Also implying its other activities, numerous mechanisms by which this vitamin epigenetically enhances genomic stability have been unveiled (reviewed in [230]). In non-malignant cells, vitamin C reduced apoptosis and downregulated 1 and upregulated 41 miRNAs in hydrogen peroxide-injured human umbilical vein endothelial cells [231]. The analysis of the 10 most changed miRNAs showed that they were involved in antiapoptotic, antioxidant, and antioncogenic protections, among other effects. In clinical studies, vitamin C intake was positively associated with the expression level of the tumor suppressors miR-31 and miR-375 in head and neck squamous cell carcinoma patients [232].

Vitamin A, in retinoic acid form, regulates gene expression through its nuclear receptors (retinoid acid receptors, RARs; and retinoid X receptors, RXRs), which are involved in DNA methylation and histone acetylation [233]. It reduces DNA methylation by upregulating TETs and promote HDACs expression by competitively displacing them from binding to retinoic acid response elements in gene promoters [201]. In addition, retinoic acid inhibits DNMTs and activates HATs [220]. The modulation DNA methylation and especially histone acetylation via retinoid receptors was particularly found to drive the important cell differentiation-inducing effect of vitamin A in stem cells [220,234]. Cell differentiation is a therapeutic target in many cancers, making retinoic acid an adopted or potential disease-modifying treatment in many of them [235,236,237]. Accordingly, a multipath effect with a great interest in cancer immunotherapy was recently unveiled. Indeed, RAR activation by vitamin A metabolites resulted, through diverse histone acetylation-related mechanisms, in the promotion of T-cell differentiation, comprehensive repression of gene expressions related to memory T-cells and, consequently, in the promotion of effector T-cell differentiation [238]. Furthermore, vitamin A modulates the expression of multiple miRNAs in healthy and malignant cells as well as in cancer progression, notably manifesting in the upregulation of many tumor-suppressing miRNAs in diverse cancers [220].

Vitamin B12 is also involved in DNA methylation by being a crucial cofactor in the methionine biosynthesis from homocysteine, given that methionine is essential in the DNA methylation process (acting as a methyl donor for methyltransferases) [201,239]. Other vitamins, such as B2, B3, B6, B9, and E, have been reported to drive diverse epigenetic regulations through diverse, and generally indirect, mechanisms, for example, being related to interference with DNA damage and repair or other biochemical pathways, although a few reports have unveiled some roles in miRNA regulation (e.g., vitamin B3) or DNA methylation (e.g., vitamin B2) in addition to the known role of some B vitamins in the methionine cycle and OCM in general (e.g., vitamins B3, B6, B9, and B12) [218,220,233,239,240]. These effects remain, however, less studied and apparently less striking than those that we have seen for other vitamins, at least according to our current level of knowledge.

Apart from methionine, which is known as a core element in methyl transfer reactions in living organisms, other amino acids such as those linked to OCM (e.g., arginine, cysteine, glycine, histidine, serine, threonine, and homocysteine) or branched-chain amino acids (leucin, isoleucine, and valine) may regulate epigenetic mechanisms in important and complex ways with direct implications in cancer and neurodegeneration pathophysiology [241,242,243,244]. Protein restrictions have also been reported to induce a global decline in DNA methylation and DNMT regulation, correlating with consequent alterations in major cell signaling pathways such as AMPK, SIRT1, and mTOR [245]. In addition, many short peptides have been shown to modulate epigenetic mechanisms and to culminate, through this modulation, in important pathophysiological roles and targeting possibilities in neurodegeneration [246,247] and cancer [247,248]. Moreover, recent evidence suggests that protein structure is also determined by epigenetic regulation and not only by amino acid sequence resulting from gene coding, as it was previously thought for decades [249]. Furthermore, we have already seen that BP is rich in proteins and almost all amino acids involved in human metabolism and that some peptides isolated from BP manifested interesting bioactivities [1]. Considering all of these observations together, we “dare” to confidently suggest that the topic of epigenetic regulatory activities must also be investigated in BP peptides. This remains a totally untapped research topic and may open new perspectives for BP-mediated epigenetic regulation, especially regarding the large number of epigenetic regulators that we have described and that exist in BPs. In addition, we take this opportunity to invite researchers to focus on the epigenetic potential of BP as a whole product or on its extracts while taking into consideration the substantial preclinical evidence that we have elucidated in this work regarding already known epigenetic modifiers that are widespread in BP.

Many experimental studies have reported that unsaturated fatty acid (omega-3, -6, and -9) supplementation have resulted in modulating global and localized DNA methylations in different cellular models, resulting in the modulation of the gene expression of diverse mediators of inflammatory and immune responses and other effects related to their anticancer potential [166,199]. Epigenetic mechanisms related to the anti-neurodegeneration potential of these fatty acids remain very scarce. Saturated fatty acids, viz., palmitate and stearate, were also found to induce the hypermethylation of isoform 1 of the peroxisome proliferator-activated receptor γ (PPARγ1) gene promoter, which is a critical determinant of pro-inflammatory activation and insulin resistance in macrophages [199]. The anti-inflammatory effect of oleic acid and other well-known fatty acids was also reported to be mediated by their modulatory effect on DNA methylation, histone modification, and microRNA signatures [250]. All of these fatty acids are present in BP, and palmitic acid is reportedly the most abundant saturated fatty acid in BP, while oleic acid is also among the main unsaturated fatty acids in this valuable matrix [1]. These are only some of the most illustrative examples, as other studies of BP fatty acids for such effects are available in the literature.

Other compounds that are frequently found in BP or that are found in high amounts in some BP types have also been reported for their important epigenetic effects. In addition to B vitamins and methionine as we have seen, **choline** is also a methyl donor in the OCM network (it is a precursor of betaine that gives the methyl group to homocysteine to be converted to methionine), therefore making DNA methylation also dependent on its presence and availability [131]. Choline is endowed with further importance as it is a key contributor to acetylcholine production and cholinergic neurotransmission, in addition to it being a crucial component of cell membranes via phosphatidylcholine production [131]. Diverse studies have reported that deficiency in dietary methyl donors may be implicated in disorders, including carcinogenesis, neurodegeneration, and other related ones such as neurodevelopmental, metabolic, and cardiovascular disorders [131,217,245,251,252]; and to results in early epigenetic alterations in offspring [253]. Accordingly, supplementation of these donors resulted in enhancing neurodevelopment and diverse neurofunctions including cognition and in reversing neurodegenerative processes in animals [245]. Specific studies have reported that supplementing choline (in animal models of AD) and betaine (in AD patients) resulted in the improvement of many NDD traits [245].

We have already seen that BP is rich in **selenium** and that normal dosage consumption of some BPs may provide the daily recommended amount of this vital element. Selenium is implicated in epigenetic regulation in an intricate and multifaceted way. It has been found to alter heterochromatin structure in murine embryonic cells, inhibit DNMTs in diverse human and animal cell lines, and induce demethylation in some cancers; meanwhile, it has acted as an HDAC inhibitor in other types of cancer [254,255,256]. These epigenetic effects have mediated anticancer effects such tumor suppressor induction and epigenetic mark deletions in many experimental studies [254]. Selenium has other epigenetic involvement phenomena, such as stabilizing telomeres and regulating homocysteine levels by contributing to SAM demethylation [255]. This essential microelement was finally reported to modulate a wide range of miRNAs, including the tumor suppressor miR-185, which was silenced in a selenium-depleted medium [254,257]. This miRNA appears, through the targeting of many genes, to play a central role in regulating carcinogenesis, progression, and metastasis and appears to affect all cancers (only a few exceptions remain to be deciphered and reported regarding the oncogenic effect of this miRNA, mainly in colorectal cancer,) [258,259]. In addition, miR-185 may be involved in mitigating neurodegeneration. In PD, where it is depleted, miR-185 has been shown to correct a great number of disease traits such as behavioral troubles, dopaminergic neuron damages, oxidative stress, and alterations of many proteins involved in PD pathophysiology [260,261].

Zinc is also markedly involved in epigenetic mechanisms. This mineral is a cornerstone element in DNA replication, repair, and transcription [245,262]. Maternal deficiency culminates in epigenetic alterations in offspring and diverse epigenetically induced diseases in later life [263], while zinc supplementation in offspring has resulted in the correction of the expression of some DNMTs and related DNA methylation levels [264]. Zinc deficiency in general results in SAM depletion and reduced DNMT function [265]. It is estimated that about 10% of human genes bind to zinc, either directly or indirectly, a binding that commonly occurs through the zinc finger domains (ZFDs), which are present in numerous transcriptional factors [262]. Zinc finger proteins are the largest group of transcription factors in mammals and are diversly involved in various cancers, either through oncogenic or tumor suppressive roles, given that they impact all main epigenetic regulations and other cancer-associated pathophysiological processes such as inflammation, apoptosis, and cancer cell proliferation and metastasis [266]. Likewise, these kinds of proteins are deeply involved in aging, neurodevelopment, brain function, and diverse neurological disorders, including neurodegeneration [267]. Indeed, many DNMTs, HATs, histone methyltransferases (HMTs), and histone demethylases carry a ZFD, which promotes their substrate access and recognition and consequently mediates their effects on gene expression, epigenetic marks, and chromatin structural changes [262,264]. Zinc-dependent HDACs are also a key group of histone deacetylases (except for sirtuins, in which zinc role remains controversial, all other known HDACs are zinc-dependent [262]) and a potential therapeutic target in cancer and neurodegeneration that is already used in clinical practice, especially in cancers [148]. Zinc deficiency increased the expression and activity of DNMT1 and DNMT3A in human cancer cell lines and upregulated DNMT1 and downregulated DNMT3A in the hippocampus of cognitive dysfunction murine models, wherein these DNMT alterations were significantly mitigated by zinc supplementation [264]. Other studies in cancer reported that zinc may inhibit DNMTs [245]. ZFD-containing proteins are also involved in demethylation, a quality that also appears to be interesting in terms of selectively reactivating genes that have been silenced by aberrant methylation in many diseases, including oncological and neurological ones [268]. For very illustrative examples, methylation-mediated silencing of miR-193b [269] and miR-128 [270] by zinc deficiency was observed in human cell lines and animal models of esophageal cancer, respectively. Interestingly, miR-193b, which was reported as a key tumor-suppressing actor in many cancers aside from some rare exceptions, was recently described as an ideal biomarker of cancer prognosis in an Asian meta-analysis. This study concluded that miR-193b expression positively correlated with poorer survival and overall prognosis in patients with diverse cancers [271]. Despite its universal alteration in neurodegeneration and cancer, the expression profile of miR-128 was controversially described in NDDs [272], whilst this expression suppressed cell proliferation, migration, invasion, and tumor growth and induced cell apoptosis in many cancers, including in the brain, where this miRNA is among the most expressed ones in humans [273]. Studies in some cancers have reported that zinc supplementation improves treatment efficiency and patient outcomes, but zinc excess is also known to result in diverse alterations such as neuroinflammation and cell toxicity [265]. These multiple and core involvements of zinc in epigenetic processes make metabolic and signaling processes in which this vital element is involved appealing targets for preventing and managing epigenetically driven diseases, especially cancers and NDDs.

Carotenoids are also a chemical family that may be endowed with important epigenetic modulatory activities. We have already discussed vitamin A for epigenetic activities, achieved mainly through retinoic acid receptors. It is therefore clear that β-carotene is importantly involved in epigenetic regulation either through DNA methylation, histone modification, or ncRNAs, given that it is metabolized to all *trans*-retinoic acid, which is extensively studied in epigenetics (it is thoroughly reviewed for cancer implications in [274], while studies in NDDs remain very scarce and inconclusive). Similar observations may be correct for β-cryptoxanthin and other provitamin A carotenoids that are present in BP. Many studies, including those in humans, have reported that β-cryptoxanthin has cancer-preventing potential independently of other carotenoids and nutraceuticals, with many effects having an epigenetic outcome (e.g., SIRT1, microbiome, and other signaling pathways), apparently resulting from the molecule itself and not from its metabolization to retinoic acid [275]. Lutein was found to increase acetyl-coenzyme A in the human undifferentiated neuroblastoma cell line [276]. Because acetyl-coenzyme A is a pivotal metabolite in epigenetic regulation by serving as supplier of the acetyl group that is transferred by HATs to lysine residues in histone [277], lutein appears to possibly have an important role in modulating histone acetylation and thus epigenetic processes. The detection of such an effect in undifferentiated cells may also open a way to therapeutic targeting of cancer cells. Nanoencapsulation has been proposed as a way to bypass the challenge of the poor stability and bioavailability of lutein for its use in therapeutics [278], a technique that is obviously promising for other natural phytochemicals. Lycopene has also been reported to downregulate DNMT3A and activate (demethylate) the gene promoter of glutathione S-transferase Pi (GSTP1, an important regulator involved in the defense against oxidative and genotoxic damages and silenced by hypermethylation in a variety of cancers [279]) in prostate cancer [280], in addition to exerting a wide range of indirect epigenetic modulations on cell signaling pathways involved in antioxidative defense, DNA damage and repair, inflammatory response, and cell death [281]. It has been also reported to upregulate (demethylate) other tumor suppressors and to be linked with the hypermethylation of possibly pro-oncogenic genes such as inflammatory mediators and T-cell activators [282,283]. However, the fact of whether this DNA methylation modulatory activity is due to DNMT inhibition or demethylation activation or both is not yet clear, and the reproducibility of the results in diverse cancers needs to be verified.

Large observational studies analyzing the six carotenoids that we have cited as examples, i.e., α-carotene, β-carotene, β-cryptoxanthin, lutein, lycopene, and zeaxanthin, have shown that circulating levels of these compounds as well as total circulating carotenoid levels were associated with reduced breast cancer risk [284]. A metabolomic analysis of these six compounds, considering their involvement in epigenetic regulations (acetylated and methylated metabolites) among other biological responses, concluded that only the metabolic signatures for β-carotene and estimated vitamin A potential significantly fulfilled the correlation with reduced breast cancer risk [284]. This conclusion supports a role of retinoic acid downstream pathways in anticancer prevention, but the extent of importance that epigenetic processes occupy in these observations were unfortunately not assessed by the authors. Due to the complexity of such an assessment, further studies are needed to decipher the real implications of epigenetic modulation by carotenoids in cancers. In fact, all carotenoid examples that we have discussed here are known to have a plethora of other cancer-mitigating bioactivities which, in spite of some very rare exceptions, are supported by a large body of evidence from preclinical and clinical studies in diverse cancers [82,285,286,287,288,289,290]. Direct studies of the implications of carotenoid-mediated epigenetic modulation in NDDs unfortunately remain a little-investigated topic, except for some studies of indirect mechanisms that we have already discussed, such as those focused on mitigating oxidative stress and DNA damage. Indeed, it may be of great significance to remember that more than 600 carotenoids are known in nature [291], leading to the prospection for these valuable compounds in BP to still be in its very early phases, especially for epigenetic interest.

Other frequent BP compounds are also known for their potential to mediate epigenetic regulations. We have already seen that BP is rich in phytosterols as it is the sole source of these vital nutrients to honeybees. Phytostanols are also widely present in plant pollens, although they are not yet studied in BP [1]. Phytosterols and phytostanols have been reported to be involved in substantially (more than two-fold) modulating more than 100 miRNAs and potently inhibiting class 1 HDAC in addition to inhibiting both the expression and activity of DNMTs [292]. β-sitosterol inhibited DNMT1 and HDAC1 overexpression and cancer cell migration and suppressed some histone methylation marks induced by hydrogen peroxide in a human breast cancer cell line [293]. Phytosterols, including β-sitosterol, activate AMPK, which is an important therapeutic target in managing many cancers [174]. This activation certainly has an epigenetic implication, at least due to the known AMPK–SIRT1 feedback loop, which is deeply involved in cancer and aging pathophysiology [294,295]. Glucosinolates have been recently reported to be widely present in BP and have even been proposed as a reliable differentiating biomarker of BP origin [296]. These compounds are known to encompass epigenetic modulating effects, and some of their derivatives such as sulforaphane are well known for their epigenetic and anticancer effects [297,298], but due to the rarity of the studies that cover their amounts in BP, we will not succinctly review them in our current work.

The human microbiota is among the major players in epigenetic regulation and in genome-related hallmarks of aging [299,300,301]. We have already elucidated its deep and complex involvement in cancer and neurodegeneration pathophysiology as well as its importance as a therapeutic target, along with the propitious potential of BP in this context. Gut microbiota for example can induce epigenetic modulation through diverse means, including the biosynthesis and metabolism of diverse methyl and acetyl donors to epigenetic-regulating enzymes; direct implication in the function and gene expression of DNMTs, HMTs, and HDACs; and modifications of diverse pathways that regulate epigenetic processes inside the host cells [300]. Gut microbiota-mediated hypermethylation of DNA was found to correlate with human disease, including metabolic and cancerous ones, and to have lifelong-lasting culminations depending on its early life profile [300]. Specific germs from the gut microbiota have also been shown to participate in immune response modulation by regulating histone modification in immune cells, a regulation that then results in impacting cytokine liberation and immune cell phenotypic changes [300]. Many long ncRNAs and some miRNAs (e.g., the tumor suppressor and inflammation regulator miR-181) have also been shown to be modulated by microbiota, while many distant microbiota-induced effects were found to be exerted through long ncRNAs, including immune response effects in distant immune organs such as the spleen and thymus [300,301]. Such implications in immune and epigenetic modulation through complex and pleiotropic ways further confirm the very important involvement of the gut microbiota that we have already elucidated in cancer and neurodegeneration pathophysiologies. There is some evidence suggesting that microbiota modulation may reverse early life epigenetic alterations, even in distant tissues. A recent study in murine models of type 1 diabetes reported that microbiota transfer from mother cecum to pups corrected the global epigenetic alterations (histone, chromatin, and miRNA changes), immune expressions, and other intestinal and distant phenotypic alterations that were induced in pups by antibiotic administration [302]. Since we have seen that BP feeding may correct microbiota alterations, a possible role of this bee cocktail in microbiota-mediated correction of epigenetic aberrances should be considered and investigated by future studies.

BP has the potential to significantly modulate the composition, diversity, abundance, and metabolites of microbiota, thus inducing both local and distant effects, as we have thoroughly explained. In addition to some metabolites that microbiota may produce, and which are known to have epigenetic roles (e.g., some polyphenols and B vitamins that we have seen), SCFAs are major “messenger” microbiota metabolites that are known to have a large plethora of local and systemic effects, including epigenetic modulation. These fatty acids are important modulators of HDAC activities and usually inhibit these deacetylases, both directly and indirectly, either locally in the gut or distantly in other organs including the CNS, generally resulting in an increased expression of target genes in addition to influencing acetyl-coenzyme A levels and methyl transfers, as well as modulating immune cell function and cytokine liberation through the modulation of related histone acetylation and miRNA signaling [300,301,303,304]. HDAC inhibition is also a key mechanism in preserving and enhancing BBB integrity by SCFAs, which manifest in reducing inflammatory aspects and enhancing epithelial cells of this barrier and manifest in a series of correcting effects on tight junctions and structural proteins [305].

Butyrate, the most potent inhibitor of HDACs among SCFAs, intricately contributes to modulating cell fate in the colon lumen through an insufficiently understood duality that depends on concentration, time, localization, and cell types [303]. It is maintained at low levels by serving as an energy source for healthy cells in colon crypts (SCFAs fulfill up to 70% of colonocyte energetic needs) and promoting the advancement of residing cells, including stem ones, through the cell cycle; meanwhile, in cancerous cells, its accumulation, due to the reliance of malignant cells on glycolysis, promotes HDAC inhibition resulting in apoptosis induction and cell proliferation suppression [303]. In the CNS, several experimental studies have reported that butyrate exerts a wide range of neurodegeneration-mitigating effects that were, at least partly, mediated by or concomitant with increased histone acetylation (reviewed in [303,306]). In cancer pathogenesis, SCFAs induce the downregulation of many oncogenic genes and reactivate the transcription of other silenced tumor suppressors in addition to diverse other anticarcinogenic effects (reviewed in [307]). SCFAs may also be involved in a large regulatory network of ncRNAs. A recent study reported that butyrate induces apoptosis and inhibits cell proliferation, invasion, and metastasis in a human colorectal cell line [308]. By adopting a computer-based prediction, this study found that butyrate acted through a complex network involving 46 miRNAs and 9 long ncRNAs. The literature on SCFAs’ effects on epigenetic processes in cancer and NDDs is very extensive, and its explanation falls beyond the scope of this review. We have just provided some elucidative examples to show the postulated potential of microbiota and SCFA modulation that may be induced by BP in epigenetic modulation.

Finally, we would like to raise a potentially important but completely untapped topic for epigenetic issues in BP. We know that extracellular vesicles (EVs) are shared communication cargos among living cells and are packed with a variety of nutrients, genetic material, and other functional molecules. Among EV load components, plant-derived miRNAs are an important element that are recently being discovered to play a major role in plant–mammalian communication and mediate, through regulating gene expression in the recipient, many physiological and/or disease-modifying roles in diverse mammal illnesses, including inflammations, cancers, and neurological alterations [309,310,311,312,313]. Plant pollen tubes bear large amounts of EVs that support their growth and ensure signal transduction and other functions [314]. Plant EVs have also been found to be excessively secreted in some pathological states of plants, such as infections [315]. In addition, EVs are widespread in bee bodies [316], and exosomes, i.e., the EVs in humans, are important epigenetic actors in many pathophysiological processes in cancers [201]. EVs have recently been characterized in BP and royal jelly for the first time [317]. This study demonstrated that BP depletion from EVs resulted in decreasing its antibacterial and biofilm inhibitory activity potential, an observation that may suggest that EVs may play important roles in other BP bioactivities. The first profiling of royal jelly miRNAs was just published and reported important results, mainly the presence of 29 known mature miRNAs and 17 novel ones, in addition to reverse ethanol-induced apoptosis and enhance cell viability [316]. The authors of this study supposed that miRNAs may be involved in the observed effects, as some of the identified miRNAs in this study were reported by diverse anterior studies to mediate observed apoptosis- and cell viability-related effects. To the best of our knowledge, there is no published study that has investigated miRNAs or other epigenetic effects of BP EVs. This appears to constitute a compelling research field and is theorized to add important perspectives to BP’s epigenetic effects.

**Figure 3:**
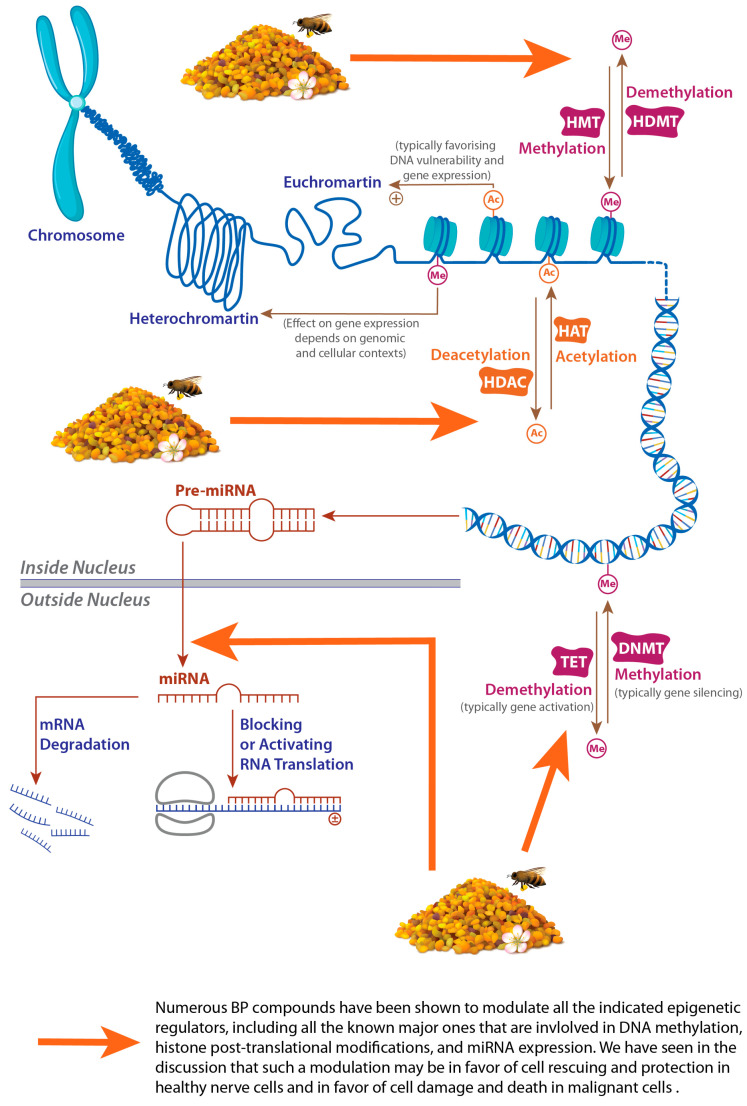
Potential mechanisms of epigenetic modulation by BP.

To conclude our discussions, Figure 3 summarizes the major effects of BP on epigenetic mechanisms that were highlighted throughout the current work.

## 4. Materials and Methods

Major scientific databases specializing in medical and pharmaceutical fields were searched for in raw scientific materials for inclusion in this review. A preliminary search was conducted in PubMed, ScienceDirect, Scopus, Web of Science, Cochrane Library, and Google Scholar. The used terms were “epigenetic “bee pollen” in Google Scholar and “epigenetic bee pollen” in other databases. The results returned were not satisfying for conducting a conclusive review, so we decided to establish a list of major BP compounds and search for their relevance to epigenetics. The compounded list that we adopted is the same as that which we adopted in our most recent review (see [2]), where we have seen in detail the importance of the chosen molecules as major BP compounds and the studies that have investigated their presence and importance in BP. To recapitulate, the chosen compounds were apigenin, catechin, chrysin, cyanidin, delphinidin, epicatechin, genistein, hesperidin, hesperetin, isorhamnetin, kaempferol, luteolin, myricetin, naringenin, naringin, pinocembrin, and quercetin for flavonoids; benzoic, caffeic, chlorogenic, cinnamic, coumaric, dihydroxybenzoic, ellagic, ferulic, gallic, hydroxycinnamic, protocatechuic, rosmarinic, syringic, and vanillic acids for phenolic acids; resveratrol for stilbene derivatives; α-carotene, β-carotene, β-cryptoxanthin, lutein, zeaxanthin, and lycopene for carotenoids; spermidine and its glycosides for phenolamides; betaine and choline for betaines; glucosinolates; and coenzyme Q10. Among BP nutrients, we mainly focused on all vitamins, minerals (copper, iron, selenium, and zinc), and phytosterols.

Firstly, we used search keywords such as “natural”, “phytochemical”, “polyphenol”, “flavonoid”, “nutrient”, and “vitamin” coupled with the keyword “epigenetic” using the “AND” Boolean function. Then, we searched for publications containing the name of the investigated molecule and the term “epigenetic”. While conducting our work, other terms such as “aging” and “age disease” were included in the search. Some recent articles were gathered to explain aging and other pathophysiological processes that we focused on in our work. The search interval timing was limited to the last five years. The initial search returned a great number of articles (more than 25 k). A total of 4387 articles were selected, classified, and analyzed to conduct our recent review and a series of reviews on BP that we will publish as soon as possible. The final number of articles used in the redaction of the current review was 327.

## 5. Concluding Remarks and Perspectives

To infer this important subsection, we can conclude that epigenetic regulation is a very promising research area in BP, and a highly complex and multifaceted potential is a priori verified for a wide spectrum of BP compounds. Because epigenetic processes are among the most studied and influential etiological factors in neurodegeneration, cancers, and age-related diseases in general, more focused studies are strongly recommended to decipher possible interventions, especially in long-term preventive interventions, but also in managing confirmed pathological states and/or in conducing prenatal interventions.

We consequently want to underline some important observations. First, it is a matter of course that some nutrient deficiencies or malnutrition in general, especially at early life stages, may have diverse and long-lasting deleterious effects on epigenetic regulation. On the other hand, maternal overnutrition and metabolic disorders are well known to be associated with substantial DNA methylation changes and other epigenetic alterations and consequent lifelong proneness in offspring to diverse disorders, including cardiometabolic, oncological and neurological diseases [318,319,320,321,322]. In addition, we have already seen that epigenetic alterations may be reversed with long-term interventions. This evidence has been verified by many preclinical studies [323,324,325,326,327], but translation into important clinical relevance is still facing diverse challenges. To correct nutritional disequilibrium and deficiencies, BP is among the best-known natural candidates.

The second observation relates to the main challenges that epigenetic research must overcome. Epigenetic regulatory processes are known to have crosstalk as one of the main characteristics. Enzymes, transcriptional factors, and diverse signaling pathways are generally involved in complex biological networks, implying diverse pathophysiological processes and interactions. In addition, age-related diseases entail very variable epigenetic signatures that may greatly differ for the same disease from one patient to another. Fortunately, due to recent advances in genomic technics, known epigenetic marks have become more easily detectable and quantifiable. Furthermore, the complexity of natural resources such as BP as well as the pleiotropic effects of sole phytocompounds such as phenolics is another challenge. These complexities render focused epigenetic targeting and avoidance of potential undesirable effects and toxicities very difficult. It is, however, encouraging that focused epigenetic interventions were possible and are authorized and successfully adopted in the clinical management of some diseases. Moreover, many studied examples of natural epigenetic modulators were reported to synergistically potentiate chemotherapeutic drugs via epigenetic mechanisms (we did not develop this issue in our work).

The third observation is that BP, as a multitarget product and a rich source of multitargeting compounds, is endowed with a valuable potential to mitigate many pathogenic processes that are usually verified as mutual culprits of epigenetic aberrances in triggering age-related pathological processes. We have thoroughly elucidated in this work and in our recent publications [1,2] that BP and its known compounds act through a multitude of mechanisms in most aging hallmarks. These data theoretically confer to BP an additional great potential in managing epigenetically induced alterations in humans. Furthermore, due to its potential safety, BP may be adopted for early and long-term interventions for epigenetic reprogramming if the research with clinical trials validates this potential.

It is also important to underline the crucial importance of resolving some major challenges that still hinder the harnessing of cumulating experimental evidence in the real world. One of the most impactful challenges is obviously the low bioavailability and bioaccessibility of natural compounds such as polyphenols and carotenoids. We have seen the example of microencapsulation as a valuable avenue in the cited studies. Future works must especially focus on novel techniques such as nano-encapsulation and synergism evaluation, in addition to establishing a deeper understanding of the pharmacodynamic and pharmacokinetic fates of natural phytocompounds, especially when delivered to human consumption in their natural matrices.

In conclusion, the availability of thousands of studies that have investigated diverse BP compounds as epigenetic modifiers is, in our opinion, a sufficient argument to conduct large-scale clinical studies, either for BP compounds, or for BP as a whole product. It is unfortunate to see that no study has been conducted in humans until now to assess the epigenetic potential of BP despite the great amount of preclinical evidence. BP is endowed with a recognized safety profile and popular consumption acceptance and should be urgently explored as a potential preventive and therapeutic arsenal to modulate age-related risks and disease course.

## Data Availability

No new data were created or analyzed in this study. Data sharing is not applicable to this article.

## References

[1] Kacemi R., Campos M.G. (2023). Translational Research on Bee Pollen as a Source of Nutrients: A Scoping Review from Bench to Real World. Nutrients.

[2] Kacemi R., Campos M.G. (2024). Translational Proofs on Bee Pollen as a Source of Biopharma-ceuticals for Neurodegeneration and Cancer Research: A Scop-ing Review and Prospective Reflections. Molecules.

[3] Kopp W. (2024). Aging and “Age-Related” Diseases—What Is the Relation?. Aging Dis..

[4] López-Otín C., Blasco M.A., Partridge L., Serrano M., Kroemer G. (2023). Hallmarks of aging: An expanding universe. Cell.

[5] Tenchov R., Sasso J.M., Wang X., Zhou Q.A. (2024). Aging Hallmarks and Progression and Age-Related Diseases: A Landscape View of Research Advancement. ACS Chem. Neurosci..

[6] Baechle J.J., Chen N., Makhijani P., Winer S., Furman D., Winer D.A. (2023). Chronic inflammation and the hallmarks of aging. Mol. Metab..

[7] Liu Z., Liang Q., Ren Y., Guo C., Ge X., Wang L., Cheng Q., Luo P., Zhang Y., Han X. (2023). Immunosenescence: Molecular mechanisms and diseases. Signal Transduct. Target. Ther..

[8] Liao S., Ning Q., Chen Y., Zhao X., Tang S. (2022). Interaction of aging and Immunosenescence: New therapeutic targets of aging. Int. Immunopharmacol..

[9] Li Y., Tian X., Luo J., Bao T., Wang S., Wu X. (2024). Molecular mechanisms of aging and anti-aging strategies. Cell Commun. Signal..

[10] Kacemi R., Campos M.G. (2023). Bee Pollen as a Source of Pharmaceuticals: Where Are We Now?. Pollen Chemistry & Biotechnology.

[11] Fard M.T., Savage K.M., Stough C.K. (2022). Peripheral inflammation marker relationships to cognition in healthy older adults—A systematic review. Psychoneuroendocrinology.

[12] Cote B., Elbarbry F., Bui F., Su J.W., Seo K., Nguyen A., Lee M., Rao D.A. (2022). Mechanistic Basis for the Role of Phytochemicals in Inflammation-Associated Chronic Diseases. Molecules.

[13] Kaur B., Singh P. (2022). Inflammation: Biochemistry, cellular targets, anti-inflammatory agents and challenges with special emphasis on cyclooxygenase-2. Bioorg. Chem..

[14] Mohammad-Rafiei F., Negahdari S., Tahershamsi Z., Gheibihayat S.M. (2024). Interface between Resolvins and Efferocytosis in Health and Disease. Cell Biochem. Biophys..

[15] Livshits G., Kalinkovich A. (2024). Restoration of epigenetic impairment in the skeletal muscle and chronic inflammation resolution as a therapeutic approach in sarcopenia. Ageing Res. Rev..

[16] Zhang J., Liu S., Ding W., Wan J., Qin J.-J., Wang M. (2024). Resolution of inflammation, an active process to restore the immune microenvironment balance: A novel drug target for treating arterial hypertension. Ageing Res. Rev..

[17] Panigrahy D., Gilligan M.M., Serhan C.N., Kashfi K. (2021). Resolution of inflammation: An organizing principle in biology and medicine. Pharmacol. Ther..

[18] Ponce J., Ulu A., Hanson C., Cameron-Smith E., Bertoni J., Wuebker J., Fisher A., Siu K.C., Marmelat V., Adamec J. (2022). Role of Specialized Pro-resolving Mediators in Reducing Neuroinflammation in Neurodegenerative Disorders. Front. Aging Neurosci..

[19] Gwak S.-Y., Kim S.-J., Park J., Kim S.H., Joe Y., Lee H.-N., Kim W., Muna I.A., Na H.-K., Chung H.T. (2022). Potential Role of Heme Oxygenase-1 in the Resolution of Experimentally Induced Colitis through Regulation of Macrophage Polarization. Gut Liver.

[20] Wang X., Li Y., Pu X., Liu G., Qin H., Wan W., Wang Y., Zhu Y., Yang J. (2024). Macrophage-related therapeutic strategies: Regulation of phenotypic switching and construction of drug delivery systems. Pharmacol. Res..

[21] Luo M., Zhao F., Cheng H., Su M., Wang Y. (2024). Macrophage polarization: An important role in inflammatory diseases. Front. Immunol..

[22] Ryyti R., Hämäläinen M., Leppänen T., Peltola R., Moilanen E. (2022). Phenolic Compounds Known to Be Present in Lingonberry (*Vaccinium vitis-idaea* L.) Enhance Macrophage Polarization towards the Anti-Inflammatory M2 Phenotype. Biomedicines.

[23] Xie K., Chai Y., Lin S., Xu F., Wang C. (2021). Luteolin Regulates the Differentiation of Regulatory T Cells and Activates IL-10-Dependent Macrophage Polarization against Acute Lung Injury. J. Immunol. Res..

[24] Fu J., Huang J., Lin M., Xie T., You T. (2020). Quercetin Promotes Diabetic Wound Healing via Switching Macrophages from M1 to M2 Polarization. J. Surg. Res..

[25] Lu B., Li C., Jing L., Zhuang F., Xiang H., Chen Y., Huang B. (2023). Rosmarinic acid nanomedicine for rheumatoid arthritis therapy: Targeted RONS scavenging and macrophage repolarization. J. Control. Release.

[26] Han D., Wu Y., Lu D., Pang J., Hu J., Zhang X., Wang Z., Zhang G., Wang J. (2023). Polyphenol-rich diet mediates interplay between macrophage-neutrophil and gut microbiota to alleviate intestinal inflammation. Cell Death Dis..

[27] Boriero D., Carcereri de Prati A., Antonini L., Ragno R., Sohji K., Mariotto S., Butturini E. (2021). The anti-STAT1 polyphenol myricetin inhibits M1 microglia activation and counteracts neuronal death. FEBS J..

[28] Ashrafizadeh M., Aref A.R., Sethi G., Ertas Y.N., Wang L. (2024). Natural product/diet-based regulation of macrophage polarization: Implications in treatment of inflammatory-related diseases and cancer. J. Nutr. Biochem..

[29] Fujiki T., Shinozaki R., Udono M., Katakura Y. (2022). Identification and Functional Evaluation of Polyphenols That Induce Regulatory T Cells. Nutrients.

[30] Poon I.K.H., Ravichandran K.S. (2024). Targeting Efferocytosis in Inflammaging. Annu. Rev. Pharmacol. Toxicol..

[31] Yao X., Liu Y., Mao M., Yang L., Zhan Q., Xiao J. (2024). Calorie restriction mimetic, resveratrol, attenuates hepatic ischemia and reperfusion injury through enhancing efferocytosis of macrophages via AMPK/STAT3/S1PR1 pathway. J. Nutr. Biochem..

[32] Li Q., Liu X., Du Y., Zhang X., Xiang P., Chen G., Ling W., Wang D. (2023). Protocatechuic acid boosts continual efferocytosis in macrophages by derepressing KLF4 to transcriptionally activate MerTK. Sci. Signal..

[33] Sears B., Saha A.K. (2021). Dietary Control of Inflammation and Resolution. Front. Nutr..

[34] Sears B., Perry M., Saha A.K. (2020). Dietary Technologies to Optimize Healing from Injury-Induced Inflammation. Antiinflamm. Antiallergy Agents Med. Chem..

[35] Fige É., Sarang Z., Sós L., Szondy Z. (2022). Retinoids Promote Mouse Bone Marrow-Derived Macrophage Differentiation and Efferocytosis via Upregulating Bone Morphogenetic Protein-2 and Smad3. Cells.

[36] Strizova Z., Benesova I., Bartolini R., Novysedlak R., Cecrdlova E., Foley L.K., Striz I. (2023). M1/M2 macrophages and their overlaps—Myth or reality?. Clin. Sci..

[37] Jamalvandi M., Khayyatzadeh S.S., Hayati M.J., Gheibihayat S.M. (2024). The role of fat-soluble vitamins in efferocytosis. Cell Biochem. Funct..

[38] Jurkovicova D., Neophytou C.M., Gašparović A.Č., Gonçalves A.C. (2022). DNA Damage Response in Cancer Therapy and Resistance: Challenges and Opportunities. Int. J. Mol. Sci..

[39] Matthews H.K., Bertoli C., de Bruin R.A.M. (2022). Cell cycle control in cancer. Nat. Rev. Mol. Cell Biol..

[40] Konopka A., Atkin J.D. (2022). The Role of DNA Damage in Neural Plasticity in Physiology and Neurodegeneration. Front. Cell. Neurosci..

[41] Zhao Y., Simon M., Seluanov A., Gorbunova V. (2023). DNA damage and repair in age-related inflammation. Nat. Rev. Immunol..

[42] Pezone A., Olivieri F., Napoli M.V., Procopio A., Avvedimento E.V., Gabrielli A. (2023). Inflammation and DNA damage: Cause, effect or both. Nat. Rev. Rheumatol..

[43] Wang H., Lautrup S., Caponio D., Zhang J., Fang E.F. (2021). DNA Damage-Induced Neurodegeneration in Accelerated Ageing and Alzheimer’s Disease. Int. J. Mol. Sci..

[44] Bordoni L., Gabbianelli R. (2020). Mitochondrial DNA and Neurodegeneration: Any Role for Dietary Antioxidants?. Antioxidants.

[45] Rong Z., Tu P., Xu P., Sun Y., Yu F., Tu N., Guo L., Yang Y. (2021). The Mitochondrial Response to DNA Damage. Front. Cell Dev. Biol..

[46] Schumacher B., Pothof J., Vijg J., Hoeijmakers J.H.J. (2021). The central role of DNA damage in the ageing process. Nature.

[47] Şahin S., Karkar B. (2019). The antioxidant properties of the chestnut bee pollen extract and its preventive action against oxidatively induced damage in DNA bases. J. Food Biochem..

[48] Chen S., Wang X., Cheng N. (2021). Ultrasound-assisted ethanol extraction of *Actinidia arguta* pollen possesses antioxidant activity and protects DNA from oxidative damage. J. Food Biochem..

[49] Bridi R., Echeverría J., Larena A., Nuñez Pizarro P., Atala E., De Camargo A.C., Oh W.Y., Shahidi F., Garcia O., Ah-Hen K.S. (2022). Honeybee Pollen From Southern Chile: Phenolic Profile, Antioxidant Capacity, Bioaccessibility, and Inhibition of DNA Damage. Front. Pharmacol..

[50] Farhan M., Rizvi A. (2022). Understanding the Prooxidant Action of Plant Polyphenols in the Cellular Microenvironment of Malignant Cells: Role of Copper and Therapeutic Implications. Front. Pharmacol..

[51] Hazafa A., Rehman K.-U.-U., Jahan N., Jabeen Z. (2020). The Role of Polyphenol (Flavonoids) Compounds in the Treatment of Cancer Cells. Nutr. Cancer.

[52] Atrahimovich D., Avni D., Khatib S. (2021). Flavonoids-macromolecules interactions in human diseases with focus on alzheimer, atherosclerosis and cancer. Antioxidants.

[53] Wang Q., Xie C., Xi S., Qian F., Peng X., Huang J., Tang F. (2020). Radioprotective effect of flavonoids on ionizing radiation-induced brain damage. Molecules.

[54] Fan X., Fan Z., Yang Z., Huang T., Tong Y., Yang D., Mao X., Yang M. (2022). Flavonoids—Natural Gifts to Promote Health and Longevity. Int. J. Mol. Sci..

[55] Costea T., Vlad O.C., Miclea L.C., Ganea C., Szöllősi J., Mocanu M.M. (2020). Alleviation of multidrug resistance by flavonoid and non-flavonoid compounds in breast, lung, colorectal and prostate cancer. Int. J. Mol. Sci..

[56] Zhang Y., Huang Y., Li Z., Wu H., Zou B., Xu Y. (2023). Exploring Natural Products as Radioprotective Agents for Cancer Therapy: Mechanisms, Challenges, and Opportunities. Cancers.

[57] Slika H., Mansour H., Wehbe N., Nasser S.A., Iratni R., Nasrallah G., Shaito A., Ghaddar T., Kobeissy F., Eid A.H. (2022). Therapeutic potential of flavonoids in cancer: ROS-mediated mechanisms. Biomed. Pharmacother..

[58] Chen X., He Z., Wu X., Mao D., Feng C., Zhang J., Chen G. (2020). Comprehensive study of the interaction between Puerariae Radix flavonoids and DNA: From theoretical simulation to structural analysis to functional analysis. Spectrochim. Acta Part A Mol. Biomol. Spectrosc..

[59] Simunkova M., Barbierikova Z., Jomova K., Hudecova L., Lauro P., Alwasel S.H., Alhazza I., Rhodes C.J., Valko M. (2021). Antioxidant vs. Prooxidant Properties of the Flavonoid, Kaempferol, in the Presence of Cu(II) Ions: A ROS-Scavenging Activity, Fenton Reaction and DNA Damage Study. Int. J. Mol. Sci..

[60] Jomova K., Hudecova L., Lauro P., Simunková M., Barbierikova Z., Malcek M., Alwasel S.H., Alhazza I.M., Rhodes C.J., Valko M. (2022). The effect of Luteolin on DNA damage mediated by a copper catalyzed Fenton reaction. J. Inorg. Biochem..

[61] Lee V.J., Heffern M.C. (2022). Structure-activity assessment of flavonoids as modulators of copper transport. Front. Chem..

[62] Kopustinskiene D.M., Jakstas V., Savickas A., Bernatoniene J. (2020). Flavonoids as Anticancer Agents. Nutrients.

[63] Sun G., Wang J., Xu X., Zhai L., Li Z., Liu J., Zhao D., Jiang R., Sun L. (2023). *Panax ginseng* Meyer cv. Silvatica phenolic acids protect DNA from oxidative damage by activating Nrf2 to protect HFF-1 cells from UVA-induced photoaging. J. Ethnopharmacol..

[64] de Abreu T.S., Braga M.A., Simão A.A., Trento M.V.C., de Faria Eleutério M.W., Silva Pereira L.L., da Cunha E.F.F., Marcussi S. (2020). Mitochondriotropic action and DNA protection: Interactions between phenolic acids and enzymes. J. Biochem. Mol. Toxicol..

[65] Majidinia M., Bishayee A., Yousefi B. (2019). Polyphenols: Major regulators of key components of DNA damage response in cancer. DNA Repair.

[66] Keyvani-Ghamsari S., Rahimi M., Khorsandi K. (2023). An update on the potential mechanism of gallic acid as an antibacterial and anticancer agent. Food Sci. Nutr..

[67] Luo L., Zhu S., Tong Y., Peng S. (2020). Ferulic Acid Induces Apoptosis of HeLa and Caski Cervical Carcinoma Cells by Down-Regulating the Phosphatidylinositol 3-Kinase (PI3K)/Akt Signaling Pathway. Med. Sci. Monit..

[68] Khalaf A.A., Hassanen E.I., Ibrahim M.A., Tohamy A.F., Aboseada M.A., Hassan H.M., Zaki A.R. (2020). Rosmarinic acid attenuates chromium-induced hepatic and renal oxidative damage and DNA damage in rats. J. Biochem. Mol. Toxicol..

[69] Zhao J., Xu L., Jin D., Xin Y., Tian L., Wang T., Zhao D., Wang Z., Wang J. (2022). Rosmarinic Acid and Related Dietary Supplements: Potential Applications in the Prevention and Treatment of Cancer. Biomolecules.

[70] Gupta A., Atanasov A.G., Li Y., Kumar N., Bishayee A. (2022). Chlorogenic acid for cancer prevention and therapy: Current status on efficacy and mechanisms of action. Pharmacol. Res..

[71] Crupi P., Faienza M.F., Naeem M.Y., Corbo F., Clodoveo M.L., Muraglia M. (2023). Overview of the Potential Beneficial Effects of Carotenoids on Consumer Health and Well-Being. Antioxidants.

[72] Terao J. (2023). Revisiting carotenoids as dietary antioxidants for human health and disease prevention. Food Funct..

[73] Bohn T., Balbuena E., Ulus H., Iddir M., Wang G., Crook N., Eroglu A. (2023). Carotenoids in Health as Studied by Omics-Related Endpoints. Adv. Nutr..

[74] González-Peña M.A., Ortega-Regules A.E., Anaya de Parrodi C., Lozada-Ramírez J.D. (2023). Chemistry, Occurrence, Properties, Applications, and Encapsulation of Carotenoids—A Review. Plants.

[75] Salazar-González C.Y., Stinco C.M., Rodríguez-Pulido F.J., Díaz-Moreno C., Fuenmayor C., Heredia F.J., González-Miret M.L. (2022). Characterization of carotenoid profile and α-tocopherol content in Andean bee pollen influenced by harvest time and particle size. LWT.

[76] Fenech M.F., Bull C.F., Van Klinken B.J.-W. (2023). Protective Effects of Micronutrient Supplements, Phytochemicals and Phytochemical-Rich Beverages and Foods Against DNA Damage in Humans: A Systematic Review of Randomized Controlled Trials and Prospective Studies. Adv. Nutr..

[77] Brahma D., Dutta D. (2023). Evaluating β-cryptoxanthin antioxidant properties against ROS-induced macromolecular damages and determining its photo-stability and in-vitro SPF. World J. Microbiol. Biotechnol..

[78] Orhan C., Tuzcu M., Gencoglu H., Sahin E., Sahin N., Ozercan I.H., Namjoshi T., Srivastava V., Morde A., Rai D. (2021). Different Doses of β-Cryptoxanthin May Secure the Retina from Photooxidative Injury Resulted from Common LED Sources. Oxidative Med. Cell. Longev..

[79] Gong Z., Platek M.E., Till C., Goodman P.J., Tangen C.M., Platz E.A., Neuhouser M.L., Thompson I.M., Santella R.M., Ambrosone C.B. (2022). Associations Between Polymorphisms in Genes Related to Oxidative Stress and DNA Repair, Interactions with Serum Antioxidants, and Prostate Cancer Risk: Results from the Prostate Cancer Prevention Trial. Front. Oncol..

[80] Zhang S.-Y., Lu Y.-Y., He X.-L., Su Y., Hu F., Wei X.-S., Pan M.-J., Zhou Q., Yang W.-B. (2023). Lutein inhibits tumor progression through the ATR/Chk1/p53 signaling pathway in non-small cell lung cancer. Phytother. Res..

[81] Marzocco S., Singla R.K., Capasso A. (2021). Multifaceted Effects of Lycopene: A Boulevard to the Multitarget-Based Treatment for Cancer. Molecules.

[82] Starska-Kowarska K. (2022). Dietary Carotenoids in Head and Neck Cancer-Molecular and Clinical Implications. Nutrients.

[83] Qi W.J., Sheng W.S., Peng C., Xiaodong M., Yao T.Z. (2021). Investigating into anti-cancer potential of lycopene: Molecular targets. Biomed. Pharmacother..

[84] Górecka D., Wawrzyniak A., Jȩdrusek-Golińska A., Dziedzic K., Hamułka J., Kowalczewski P.Ł., Walkowiak J., Jędrusek-Golińska A., Dziedzic K., Hamułka J. (2020). Lycopene in tomatoes and tomato products. Open Chem..

[85] Estevinho L.M., Dias T., Anjos O. (2019). Influence of the Storage Conditions (Frozen vs. Dried) in Health-Related Lipid Indexes and Antioxidants of Bee Pollen. Eur. J. Lipid Sci. Technol..

[86] Rong Y., Mi X., Ni C., Liu T., Yang N., Hong J., Li Y., Li Z., Han D., Guo X. (2022). Protective effect of vitamin C on DNA damage in surgery-induced cognitive dysfunction in APP/PS1 mice. Neurosci. Lett..

[87] Varesi A., Chirumbolo S., Campagnoli L.I.M., Pierella E., Piccini G.B., Carrara A., Ricevuti G., Scassellati C., Bonvicini C., Pascale A. (2022). The Role of Antioxidants in the Interplay between Oxidative Stress and Senescence. Antioxidants.

[88] Mascolo E., Liguori F., Merigliano C., Schiano L., Gnocchini E., Pilesi E., Volonté C., Di Salvo M.L., Contestabile R., Tramonti A. (2022). Vitamin B6 rescues insulin resistance and glucose-induced DNA damage caused by reduced activity of *Drosophila* PI3K. J. Cell. Physiol..

[89] Contestabile R., di Salvo M.L., Bunik V., Tramonti A., Vernì F. (2020). The multifaceted role of vitamin B6 in cancer: *Drosophila* as a model system to investigate DNA damage. Open Biol..

[90] Li Z., Cai K., Sun Y., Zhou D., Yan J., Luo S., Huang G., Gao Y., Li W. (2023). Folic acid protects against age-associated apoptosis and telomere attrition of neural stem cells in senescence-accelerated mouse prone 8. Appl. Physiol. Nutr. Metab..

[91] Li Z., Li W., Zhou D., Zhao J., Ma Y., Huang L., Dong C., Wilson J.X., Huang G. (2022). Alleviating Oxidative Damage–Induced Telomere Attrition: A Potential Mechanism for Inhibition by Folic Acid of Apoptosis in Neural Stem Cells. Mol. Neurobiol..

[92] de Lima-Reis S.R., Silva T.A., Costa L.S.A., Volp A.C.P., Rios-Santos F., Reis É.M., Bassi-Branco C.L. (2022). Serum levels of vitamin A, selenium, and better dietary total antioxidant capacity are related to lower oxidative DNA damage: A cross-sectional study of individuals at cardiovascular risk. J. Nutr. Biochem..

[93] Halczuk K., Kaźmierczak-Barańska J., Karwowski B.T., Karmańska A., Cieślak M. (2023). Vitamin B12-Multifaceted In Vivo Functions and In Vitro Applications. Nutrients.

[94] Doostabadi M.R., Hassanzadeh-Taheri M., Asgharzadeh M., Mohammadzadeh M. (2021). Protective effect of vitamin e on sperm parameters, chromatin quality, and dna fragmentation in mice treated with different doses of ethanol: An experimental study. Int. J. Reprod. Biomed..

[95] Costa M.I., Lapa B.S., Jorge J., Alves R., Carreira I.M., Sarmento-Ribeiro A.B., Gonçalves A.C. (2022). Zinc Prevents DNA Damage in Normal Cells but Shows Genotoxic and Cytotoxic Effects in Acute Myeloid Leukemia Cells. Int. J. Mol. Sci..

[96] Sahu C., Dwivedi D.K., Jena G.B. (2020). Zinc and selenium combination treatment protected diabetes-induced testicular and epididymal damage in rat. Hum. Exp. Toxicol..

[97] İpek E., Hesapçıoğlu M., Karaboğa M., Avcı H. (2023). Selenium protection from DNA damage and regulation of apoptosis signaling following cyclophosphamide induced cardiotoxicity in rats. Biotech. Histochem..

[98] Ahsan A., Liu Z., Su R., Liu C., Liao X., Su M. (2022). Potential Chemotherapeutic Effect of Selenium for Improved Canceration of Esophageal Cancer. Int. J. Mol. Sci..

[99] Kim S.J., Choi M.C., Park J.M., Chung A.S. (2021). Antitumor Effects of Selenium. Int. J. Mol. Sci..

[100] Didier A.J., Stiene J., Fang L., Watkins D., Dworkin L.D., Creeden J.F. (2023). Antioxidant and Anti-Tumor Effects of Dietary Vitamins A, C, and E. Antioxidants.

[101] Fagbohun O.F., Gillies C.R., Murphy K.P.J., Rupasinghe H.P.V. (2023). Role of Antioxidant Vitamins and Other Micronutrients on Regulations of Specific Genes and Signaling Pathways in the Prevention and Treatment of Cancer. Int. J. Mol. Sci..

[102] Rai S.N., Singh P., Steinbusch H.W.M., Vamanu E., Ashraf G., Singh M.P. (2021). The Role of Vitamins in Neurodegenerative Disease: An Update. Biomedicines.

[103] Shah H., Dehghani F., Ramezan M., Gannaban R.B., Haque Z.F., Rahimi F., Abbasi S., Shin A.C. (2023). Revisiting the Role of Vitamins and Minerals in Alzheimer’s Disease. Antioxidants.

[104] Constantinou C., Charalambous C., Kanakis D. (2020). Vitamin E and cancer: An update on the emerging role of γ and δ tocotrienols. Eur. J. Nutr..

[105] Kang M., Park S., Park S.-H., Lee H.G., Park J.H. (2022). A Double-Edged Sword: The Two Faces of PARylation. Int. J. Mol. Sci..

[106] Maluchenko N.V., Feofanov A.V., Studitsky V.M. (2021). PARP-1-Associated Pathological Processes: Inhibition by Natural Polyphenols. Int. J. Mol. Sci..

[107] Zhang J., Zhang J., Li H., Chen L., Yao D. (2023). Dual-target inhibitors of PARP1 in cancer therapy: A drug discovery perspective. Drug Discov. Today.

[108] Thapa K., Khan H., Sharma U., Grewal A.K., Singh T.G. (2021). Poly (ADP-ribose) polymerase-1 as a promising drug target for neurodegenerative diseases. Life Sci..

[109] Maggiore A., Casale A.M., Toscanelli W., Cappucci U., Rotili D., Grieco M., Gagné J.P., Poirier G.G., D’erme M., Piacentini L. (2022). Neuroprotective Effects of PARP Inhibitors in *Drosophila* Models of Alzheimer’s Disease. Cells.

[110] BinMowyna M.N., AlFaris N.A. (2021). Kaempferol suppresses acetaminophen-induced liver damage by upregulation/activation of SIRT1. Pharm. Biol..

[111] Li Y., Fan B., Pu N., Ran X., Lian T., Cai Y., Xing W., Sun K. (2022). Isorhamnetin Suppresses Human Gastric Cancer Cell Proliferation through Mitochondria-Dependent Apoptosis. Molecules.

[112] Xi X., Li J., Guo S., Li Y., Xu F., Zheng M., Cao H., Cui X., Guo H., Han C. (2018). The Potential of Using Bee Pollen in Cosmetics: A Review. J. Oleo Sci..

[113] Salech F., Ponce D.P., Paula-Lima A.C., SanMartin C.D., Behrens M.I. (2020). Nicotinamide, a Poly [ADP-Ribose] Polymerase 1 (PARP-1) Inhibitor, as an Adjunctive Therapy for the Treatment of Alzheimer’s Disease. Front. Aging Neurosci..

[114] Kumar V., Kumar A., Mir K.U.I., Yadav V., Chauhan S.S. (2022). Pleiotropic role of PARP1: An overview. 3 Biotech.

[115] Rossiello F., Jurk D., Passos J.F., d’Adda di Fagagna F. (2022). Telomere dysfunction in ageing and age-related diseases. Nat. Cell Biol..

[116] Ye Q., Apsley A.T., Etzel L., Hastings W.J., Kozlosky J.T., Walker C., Wolf S.E., Shalev I. (2023). Telomere length and chronological age across the human lifespan: A systematic review and meta-analysis of 414 study samples including 743,019 individuals. Ageing Res. Rev..

[117] Saretzki G., Wan T. (2021). Telomerase in Brain: The New Kid on the Block and Its Role in Neurodegenerative Diseases. Biomedicines.

[118] Rysz J., Franczyk B., Rysz-Górzyńska M., Gluba-Brzózka A. (2022). Ageing, age-related cardiovascular risk and the beneficial role of natural components intake. Int. J. Mol. Sci..

[119] Sorrenti V., Buriani A., Fortinguerra S., Davinelli S., Scapagnini G., Cassidy A., De Vivo I. (2023). Cell Survival, Death, and Proliferation in Senescent and Cancer Cells: The Role of (Poly)phenols. Adv. Nutr..

[120] Cho S.J., Pronko A., Yang J., Stout-Delgado H. (2023). Impact of Senolytic Treatment on Gene Expression in Aged Lung. Int. J. Mol. Sci..

[121] Mostafa H., Gutierrez-Tordera L., Mateu-Fabregat J., Papandreou C., Bulló M. (2024). Dietary fat, telomere length and cognitive function: Unravelling the complex relations. Curr. Opin. Lipidol..

[122] Zhou D., Sun Y., Dong C., Wang Z., Zhao J., Li Z., Huang G., Li W. (2024). Folic acid alleviated oxidative stress-induced telomere attrition and inhibited apoptosis of neurocytes in old rats. Eur. J. Nutr..

[123] Maleki M., Khelghati N., Alemi F., Bazdar M., Asemi Z., Majidinia M., Sadeghpoor A., Mahmoodpoor A., Jadidi-Niaragh F., Targhazeh N. (2020). Stabilization of telomere by the antioxidant property of polyphenols: Anti-aging potential. Life Sci..

[124] Kaźmierczak-Barańska J., Boguszewska K., Karwowski B.T. (2020). Nutrition Can Help DNA Repair in the Case of Aging. Nutrients.

[125] Gutlapalli S.D., Kondapaneni V., Toulassi I.A., Poudel S., Zeb M., Choudhari J., Cancarevic I. (2020). The Effects of Resveratrol on Telomeres and Post Myocardial Infarction Remodeling. Cureus.

[126] Teramoto N., Okada Y., Aburada N., Hayashi M., Ito J., Shirasuna K., Iwata H. (2024). Resveratrol intake by males increased the mitochondrial DNA copy number and telomere length of blastocysts derived from aged mice. J. Reprod. Dev..

[127] Dion C., Laberthonnière C., Magdinier F. (2023). Épigénétique, principes et exemples d’applications. Rev. Méd. Interne.

[128] Farsetti A., Illi B., Gaetano C. (2023). How epigenetics impacts on human diseases. Eur. J. Intern. Med..

[129] Carlberg C., Velleuer E. (2023). Nutrition and epigenetic programming. Curr. Opin. Clin. Nutr. Metab. Care.

[130] Mohd Murshid N., Aminullah Lubis F., Makpol S. (2022). Epigenetic Changes and Its Intervention in Age-Related Neurodegenerative Diseases. Cell. Mol. Neurobiol..

[131] Bekdash R.A. (2023). Methyl Donors, Epigenetic Alterations, and Brain Health: Understanding the Connection. Int. J. Mol. Sci..

[132] Li X., Qi L. (2022). Epigenetics in Precision Nutrition. J. Pers. Med..

[133] Simpson D.J., Chandra T. (2021). Epigenetic age prediction. Aging Cell.

[134] He X., Liu J., Liu B., Shi J. (2021). The use of DNA methylation clock in aging research. Exp. Biol. Med..

[135] Salameh Y., Bejaoui Y., El Hajj N. (2020). DNA Methylation Biomarkers in Aging and Age-Related Diseases. Front. Genet..

[136] Loganathan T., Doss C.G.P. (2023). Non-coding RNAs in human health and disease: Potential function as biomarkers and therapeutic targets. Funct. Integr. Genom..

[137] Olufunmilayo E.O., Holsinger R.M.D. (2023). Roles of Non-Coding RNA in Alzheimer’s Disease Pathophysiology. Int. J. Mol. Sci..

[138] Pekarek L., Torres-Carranza D., Fraile-Martinez O., García-Montero C., Pekarek T., Saez M.A., Rueda-Correa F., Pimentel-Martinez C., Guijarro L.G., Diaz-Pedrero R. (2023). An Overview of the Role of MicroRNAs on Carcinogenesis: A Focus on Cell Cycle, Angiogenesis and Metastasis. Int. J. Mol. Sci..

[139] Morenikeji O.B., Kutchy N.A. (2023). Editorial: Role of non-coding RNAs, metabolites, and extracellular vesicles in disease regulation and health. Front. Genet..

[140] Recillas-Targa F. (2022). Cancer Epigenetics: An Overview. Arch. Med. Res..

[141] Castro-Muñoz L.J., Ulloa E.V., Sahlgren C., Lizano M., De La Cruz-Hernández E., Contreras-Paredes A. (2023). Modulating epigenetic modifications for cancer therapy (Review). Oncol. Rep..

[142] Lyubitelev A., Studitsky V. (2023). Inhibition of Cancer Development by Natural Plant Polyphenols: Molecular Mechanisms. Int. J. Mol. Sci..

[143] Xie J., Xie L., Wei H., Li X.-J.J., Lin L. (2023). Dynamic Regulation of DNA Methylation and Brain Functions. Biology.

[144] Jiang S., Guo Y. (2020). Epigenetic Clock: DNA Methylation in Aging. Stem Cells Int..

[145] Kabir F., Atkinson R., Cook A.L., Phipps A.J., King A.E. (2023). The role of altered protein acetylation in neurodegenerative disease. Front. Aging Neurosci..

[146] Ghosh P., Saadat A. (2023). Neurodegeneration and epigenetics: A review. Neurología.

[147] Rodrigues D.A., Pinheiro P.D.S.M., Sagrillo F.S., Bolognesi M.L., Fraga C.A.M. (2020). Histone deacetylases as targets for the treatment of neurodegenerative disorders: Challenges and future opportunities. Med. Res. Rev..

[148] Li Y., Lin S., Gu Z., Chen L., He B. (2022). Zinc-dependent deacetylases (HDACs) as potential targets for treating Alzheimer’s disease. Bioorg. Med. Chem. Lett..

[149] Giallongo S., Longhitano L., Denaro S., D’Aprile S., Torrisi F., La Spina E., Giallongo C., Mannino G., Lo Furno D., Zappalà A. (2022). The Role of Epigenetics in Neuroinflammatory-Driven Diseases. Int. J. Mol. Sci..

[150] Dai Y., Wei T., Shen Z., Bei Y., Lin H., Dai H. (2021). Classical HDACs in the regulation of neuroinflammation. Neurochem. Int..

[151] Toker L., Tran G.T., Sundaresan J., Tysnes O.B., Alves G., Haugarvoll K., Nido G.S., Dölle C., Tzoulis C. (2021). Genome-wide histone acetylation analysis reveals altered transcriptional regulation in the Parkinson’s disease brain. Mol. Neurodegener..

[152] Nativio R., Lan Y., Donahue G., Sidoli S., Berson A., Srinivasan A.R., Shcherbakova O., Amlie-Wolf A., Nie J., Cui X. (2020). An integrated multi-omics approach identifies epigenetic alterations associated with Alzheimer’s disease. Nat. Genet..

[153] Hahn A., Pensold D., Bayer C., Tittelmeier J., González-Bermúdez L., Marx-Blümel L., Linde J., Groß J., Salinas-Riester G., Lingner T. (2020). DNA Methyltransferase 1 (DNMT1) Function Is Implicated in the Age-Related Loss of Cortical Interneurons. Front. Cell Dev. Biol..

[154] Berry K.P., Lu Q.R. (2020). Chromatin modification and epigenetic control in functional nerve regeneration. Semin. Cell Dev. Biol..

[155] Zaib S., Rana N., Khan I. (2022). Histone Modifications and their Role in Epigenetics of Cancer. Curr. Med. Chem..

[156] Dang F., Wei W. (2022). Targeting the acetylation signaling pathway in cancer therapy. Semin. Cancer Biol..

[157] Pandey P., Khan F., Seifeldin S.A., Alshaghdali K., Siddiqui S., Abdelwadoud M.E., Vyas M., Saeed M., Mazumder A., Saeed A. (2023). Targeting Wnt/β-Catenin Pathway by Flavonoids: Implication for Cancer Therapeutics. Nutrients.

[158] Ramakrishna K., Nalla L.V., Naresh D., Venkateswarlu K., Viswanadh M.K., Nalluri B.N., Chakravarthy G., Duguluri S., Singh P., Rai S.N. (2023). WNT-β Catenin Signaling as a Potential Therapeutic Target for Neurodegenerative Diseases: Current Status and Future Perspective. Diseases.

[159] Sharma A., Mir R., Galande S. (2021). Epigenetic Regulation of the Wnt/β-Catenin Signaling Pathway in Cancer. Front. Genet..

[160] Hayat R., Manzoor M., Hussain A. (2022). Wnt signaling pathway: A comprehensive review. Cell Biol. Int..

[161] Zhang X., Yu X. (2023). Crosstalk between Wnt/β-catenin signaling pathway and DNA damage response in cancer: A new direction for overcoming therapy resistance. Front. Pharmacol..

[162] Rajendran P., Abdelsalam S.A., Renu K., Veeraraghavan V., Ben Ammar R., Ahmed E.A. (2022). Polyphenols as Potent Epigenetics Agents for Cancer. Int. J. Mol. Sci..

[163] Ghasemi S., Xu S., Nabavi S.M., Amirkhani M.A., Sureda A., Tejada S., Lorigooini Z. (2021). Epigenetic targeting of cancer stem cells by polyphenols (cancer stem cells targeting). Phytother. Res..

[164] Pereira Q.C., Dos Santos T.W., Fortunato I.M., Ribeiro M.L. (2023). The Molecular Mechanism of Polyphenols in the Regulation of Ageing Hallmarks. Int. J. Mol. Sci..

[165] Kumari A., Bhawal S., Kapila S., Yadav H., Kapila R. (2022). Health-promoting role of dietary bioactive compounds through epigenetic modulations: A novel prophylactic and therapeutic approach. Crit. Rev. Food Sci. Nutr..

[166] Milošević M., Arsić A., Cvetković Z., Vučić V. (2021). Memorable Food: Fighting Age-Related Neurodegeneration by Precision Nutrition. Front. Nutr..

[167] Zeng Y., Zhang J., Yue J., Han G., Liu W., Liu L., Lin X., Zha Y., Liu J., Tan Y. (2022). The Role of DACT Family Members in Tumorigenesis and Tumor Progression. Int. J. Biol. Sci..

[168] Fatima N., Baqri S.S.R., Bhattacharya A., Koney N.K.-K., Husain K., Abbas A., Ansari R.A. (2021). Role of Flavonoids as Epigenetic Modulators in Cancer Prevention and Therapy. Front. Genet..

[169] Khan H., Belwal T., Efferth T., Farooqi A.A., Sanches-Silva A., Vacca R.A., Nabavi S.F.M., Khan F., Prasad Devkota H., Barreca D. (2021). Targeting epigenetics in cancer: Therapeutic potential of flavonoids. Crit. Rev. Food Sci. Nutr..

[170] Wu H., Cui M., Li C., Li H., Dai Y., Cui K., Li Z. (2021). Kaempferol Reverses Aerobic Glycolysis via miR-339-5p-Mediated PKM Alternative Splicing in Colon Cancer Cells. J. Agric. Food Chem..

[171] Torello C.O., Alvarez M.C., Olalla Saad S.T. (2021). Polyphenolic Flavonoid Compound Quercetin Effects in the Treatment of Acute Myeloid Leukemia and Myelodysplastic Syndromes. Molecules.

[172] Selvakumar P., Badgeley A., Murphy P., Anwar H., Sharma U., Lawrence K., Lakshmikuttyamma A. (2020). Flavonoids and Other Polyphenols Act as Epigenetic Modifiers in Breast Cancer. Nutrients.

[173] Zhang L., Kang Q., Kang M., Jiang S., Yang F., Gong J., Ou G., Wang S. (2023). Regulation of main ncRNAs by polyphenols: A novel anticancer therapeutic approach. Phytomedicine.

[174] Bhattacharya T., Dutta S., Akter R., Rahman M.H., Karthika C., Nagaswarupa H.P., Murthy H.C.A., Fratila O., Brata R., Bungau S. (2021). Role of Phytonutrients in Nutrigenetics and Nutrigenomics Perspective in Curing Breast Cancer. Biomolecules.

[175] Quiñonero F., Mesas C., Peña M., Cabeza L., Perazzoli G., Melguizo C., Ortiz R., Prados J. (2023). Vegetal-Derived Bioactive Compounds as Multidrug Resistance Modulators in Colorectal Cancer. Appl. Sci..

[176] Leri M., Scuto M., Ontario M.L., Calabrese V., Calabrese E.J., Bucciantini M., Stefani M. (2020). Healthy Effects of Plant Polyphenols: Molecular Mechanisms. Int. J. Mol. Sci..

[177] Da Costa P.C.T., de Souza E.L., Lacerda D.C., Cruz Neto J.P.R., de Sales L.C.S., Silva Luis C.C., Pontes P.B., Cavalcanti Neto M.P., de Brito Alves J.L. (2022). Evidence for Quercetin as a Dietary Supplement for the Treatment of Cardio-Metabolic Diseases in Pregnancy: A Review in Rodent Models. Foods.

[178] Arias C., Salazar L.A. (2022). Autophagy and polyphenols in osteoarthritis: A focus on epigenetic regulation. Int. J. Mol. Sci..

[179] Griñán-Ferré C., Bellver-Sanchis A., Izquierdo V., Corpas R., Roig-Soriano J., Chillón M., Andres-Lacueva C., Somogyvári M., Sőti C., Sanfeliu C. (2021). The pleiotropic neuroprotective effects of resveratrol in cognitive decline and Alzheimer’s disease pathology: From antioxidant to epigenetic therapy. Ageing Res. Rev..

[180] Levenson A.S. (2022). Metastasis-associated protein 1-mediated antitumor and anticancer activity of dietary stilbenes for prostate cancer chemoprevention and therapy. Semin. Cancer Biol..

[181] Fortunato I.M., Dos Santos T.W., Ferraz L.F.C., Santos J.C., Ribeiro M.L. (2021). Effect of Polyphenols Intake on Obesity-Induced Maternal Programming. Nutrients.

[182] Pontes P.B., Toscano A.E., Lacerda D.C., da Silva Araújo E.R., da Costa P.C.T., Alves S.M., de Brito Alves J.L., Manhães-de-Castro R. (2023). Effectiveness of Polyphenols on Perinatal Brain Damage: A Systematic Review of Preclinical Studies. Foods.

[183] Pyo I.S., Yun S., Yoon Y.E., Choi J.W., Lee S.J. (2020). Mechanisms of aging and the preventive effects of resveratrol on age-related diseases. Molecules.

[184] Ungurianu A., Zanfirescu A., Margină D. (2023). Sirtuins, resveratrol and the intertwining cellular pathways connecting them. Ageing Res. Rev..

[185] Razick D.I., Akhtar M., Wen J., Alam M., Dean N., Karabala M., Ansari U., Ansari Z., Tabaie E., Siddiqui S. (2023). The Role of Sirtuin 1 (SIRT1) in Neurodegeneration. Cureus.

[186] Ku H.C., Cheng C.F. (2020). Master Regulator Activating Transcription Factor 3 (ATF3) in Metabolic Homeostasis and Cancer. Front. Endocrinol..

[187] Javed Z., Sadia H., Iqbal M.J., Shamas S., Malik K., Ahmed R., Raza S., Butnariu M., Cruz-Martins N., Sharifi-Rad J. (2021). Apigenin role as cell-signaling pathways modulator: Implications in cancer prevention and treatment. Cancer Cell Int..

[188] Husain K., Villalobos-Ayala K., Laverde V., Vazquez O.A., Miller B., Kazim S., Blanck G., Hibbs M.L., Krystal G., Elhussin I. (2022). Apigenin Targets MicroRNA-155, Enhances SHIP-1 Expression, and Augments Anti-Tumor Responses in Pancreatic Cancer. Cancers.

[189] Wang S.-M., Yang P.-W., Feng X.-J., Zhu Y.-W., Qiu F.-J., Hu X.-D., Zhang S.-H. (2021). Apigenin Inhibits the Growth of Hepatocellular Carcinoma Cells by Affecting the Expression of microRNA Transcriptome. Front. Oncol..

[190] Singh Tuli H., Rath P., Chauhan A., Sak K., Aggarwal D., Choudhary R., Sharma U., Vashishth K., Sharma S., Kumar M. (2022). Luteolin, a Potent Anticancer Compound: From Chemistry to Cellular Interactions and Synergetic Perspectives. Cancers.

[191] Madureira M.B., Concato V.M., Cruz E.M.S., Bitencourt de Morais J.M., Inoue F.S.R., Concimo Santos N., Gonçalves M.D., Cremer de Souza M., Basso Scandolara T., Fontana Mezoni M. (2023). Naringenin and Hesperidin as Promising Alternatives for Prevention and Co-Adjuvant Therapy for Breast Cancer. Antioxidants.

[192] Chen B., Zhang W., Lin C., Zhang L. (2022). A Comprehensive Review on Beneficial Effects of Catechins on Secondary Mitochondrial Diseases. Int. J. Mol. Sci..

[193] Proshkina E., Shaposhnikov M., Moskalev A. (2020). Genome-Protecting Compounds as Potential Geroprotectors. Int. J. Mol. Sci..

[194] Baky M.H., Abouelela M.B., Wang K., Farag M.A. (2023). Bee Pollen and Bread as a Super-Food: A Comparative Review of Their Metabolome Composition and Quality Assessment in the Context of Best Recovery Conditions. Molecules.

[195] Thakur M., Nanda V. (2020). Composition and functionality of bee pollen: A review. Trends Food Sci. Technol..

[196] El Ghouizi A., Bakour M., Laaroussi H., Ousaaid D., El Menyiy N., Hano C., Lyoussi B. (2023). Bee Pollen as Functional Food: Insights into Its Composition and Therapeutic Properties. Antioxidants.

[197] Li Y., Chen F., Wei A., Bi F., Zhu X., Yin S., Lin W., Cao W. (2019). Klotho recovery by genistein via promoter histone acetylation and DNA demethylation mitigates renal fibrosis in mice. J. Mol. Med..

[198] Sorrenti V., Fortinguerra S., Caudullo G., Buriani A. (2020). Deciphering the role of polyphenols in sports performance: From nutritional genomics to the gut microbiota toward phytonutritional epigenomics. Nutrients.

[199] Ramos-Lopez O., Milagro F.I., Riezu-Boj J.I., Martinez J.A. (2021). Epigenetic signatures underlying inflammation: An interplay of nutrition, physical activity, metabolic diseases, and environmental factors for personalized nutrition. Inflamm. Res..

[200] Borsoi F.T., Neri-Numa I.A., de Oliveira W.Q., de Araújo F.F., Pastore G.M. (2023). Dietary polyphenols and their relationship to the modulation of non-communicable chronic diseases and epigenetic mechanisms: A mini-review. Food Chem. Mol. Sci..

[201] Rubio K., Hernández-Cruz E.Y., Rogel-Ayala D.G., Sarvari P., Isidoro C., Barreto G., Pedraza-Chaverri J. (2023). Nutriepigenomics in Environmental-Associated Oxidative Stress. Antioxidants.

[202] Divyajanani S., Harithpriya K., Ganesan K., Ramkumar K.M. (2023). Dietary Polyphenols Remodel DNA Methylation Patterns of NRF2 in Chronic Disease. Nutrients.

[203] Lagoa R., Marques-da-Silva D., Diniz M., Daglia M., Bishayee A. (2022). Molecular mechanisms linking environmental toxicants to cancer development: Significance for protective interventions with polyphenols. Semin. Cancer Biol..

[204] Yessenkyzy A., Saliev T., Zhanaliyeva M., Masoud A.-R., Umbayev B., Sergazy S., Krivykh E., Gulyayev A., Nurgozhin T. (2020). Polyphenols as Caloric-Restriction Mimetics and Autophagy Inducers in Aging Research. Nutrients.

[205] Arora I., Sharma M., Sun L.Y., Tollefsbol T.O. (2020). The epigenetic link between polyphenols, aging and age-related diseases. Genes.

[206] Roy B., Lee E., Li T., Rampersaud M. (2022). Role of miRNAs in Neurodegeneration: From Disease Cause to Tools of Biomarker Discovery and Therapeutics. Genes.

[207] Baby N., Alagappan N., Dheen S.T., Sajikumar S. (2020). MicroRNA-134-5p inhibition rescues long-term plasticity and synaptic tagging/capture in an Aβ(1–42)-induced model of Alzheimer’s disease. Aging Cell.

[208] Morris G., Reschke C.R., Henshall D.C. (2019). Targeting microRNA-134 for seizure control and disease modification in epilepsy. eBioMedicine.

[209] Bahlakeh G., Gorji A., Soltani H., Ghadiri T. (2021). MicroRNA alterations in neuropathologic cognitive disorders with an emphasis on dementia: Lessons from animal models. J. Cell. Physiol..

[210] Pan J.-Y., Zhang F., Sun C.-C., Li S.-J., Li G., Gong F.-Y., Bo T., He J., Hua R.-X., Hu W.-D. (2017). miR-134: A Human Cancer Suppressor?. Mol. Ther. Nucleic Acids.

[211] Ma Z., Li K., Chen P., Pan Q., Li X., Zhao G. (2020). MiR-134, Mediated by IRF1, Suppresses Tumorigenesis and Progression by Targeting VEGFA and MYCN in Osteosarcoma. Anticancer. Agents Med. Chem..

[212] Abozaid O.A.R., Sallam M.W., El-Sonbaty S., Aziza S., Emad B., Ahmed E.S.A. (2022). Resveratrol-Selenium Nanoparticles Alleviate Neuroinflammation and Neurotoxicity in a Rat Model of Alzheimer’s Disease by Regulating Sirt1/miRNA-134/GSK3β Expression. Biol. Trace Elem. Res..

[213] Korać P., Antica M., Matulić M. (2021). Mir-7 in cancer development. Biomedicines.

[214] Zhu S., Choudhury N.R., Rooney S., Pham N.T., Koszela J., Kelly D., Spanos C., Rappsilber J., Auer M., Michlewski G. (2021). RNA pull-down confocal nanoscanning (RP-CONA) detects quercetin as pri-miR-7/HuR interaction inhibitor that decreases α-synuclein levels. Nucleic Acids Res..

[215] Zhang J., Zhao M., Yan R., Liu J., Maddila S., Junn E., Mouradian M.M. (2021). MicroRNA-7 Protects Against Neurodegeneration Induced by α-Synuclein Preformed Fibrils in the Mouse Brain. Neurotherapeutics.

[216] Mondal P., Natesh J., Penta D., Meeran S.M. (2022). Progress and promises of epigenetic drugs and epigenetic diets in cancer prevention and therapy: A clinical update. Semin. Cancer Biol..

[217] Ghazi T., Arumugam T., Foolchand A., Chuturgoon A.A. (2020). The Impact of Natural Dietary Compounds and Food-Borne Mycotoxins on DNA Methylation and Cancer. Cells.

[218] Gómez de Cedrón M., Moreno Palomares R., Ramírez de Molina A. (2023). Metabolo-epigenetic interplay provides targeted nutritional interventions in chronic diseases and ageing. Front. Oncol..

[219] Sharma S., Bhonde R. (2023). Epigenetic Modifiers as Game Changers for Healthy Aging. Rejuvenation Res..

[220] Nur S.M., Rath S., Ahmad V., Ahmad A., Ateeq B., Khan M.I. (2021). Nutritive vitamins as epidrugs. Crit. Rev. Food Sci. Nutr..

[221] Barrero M.J., Cejas P., Long H.W., Ramirez de Molina A. (2022). Nutritional Epigenetics in Cancer. Adv. Nutr..

[222] Holzapfel C., Waldenberger M., Lorkowski S., Daniel H., Working Group “Personalized Nutrition” of the German Nutrition Society (2022). Genetics and Epigenetics in Personalized Nutrition: Evidence, Expectations, and Experiences. Mol. Nutr. Food Res..

[223] Siddeek B., Simeoni U. (2022). Epigenetics provides a bridge between early nutrition and long-term health and a target for disease prevention. Acta Paediatr. Int. J. Paediatr..

[224] Vetter V.M., Sommerer Y., Kalies C.H., Spira D., Bertram L., Demuth I. (2022). Vitamin D supplementation is associated with slower epigenetic aging. GeroScience.

[225] Snegarova V., Naydenova D. (2020). Vitamin D: A Review of its Effects on Epigenetics and Gene Regulation. Folia Med..

[226] Pavlovic V., Ciric M., Petkovic M., Golubovic M. (2023). Vitamin C and epigenetics: A short physiological overview. Open Med..

[227] Maity J., Majumder S., Pal R., Saha B., Mukhopadhyay P.K. (2023). Ascorbic acid modulates immune responses through Jumonji-C domain containing histone demethylases and Ten eleven translocation (TET) methylcytosine dioxygenase. Bioessays.

[228] Zhang X., Zhang Y., Wang C., Wang X. (2023). TET (Ten-eleven translocation) family proteins: Structure, biological functions and applications. Signal Transduct. Target. Ther..

[229] Yang J., Hu Y., Zhang B., Liang X., Li X. (2022). The JMJD Family Histone Demethylases in Crosstalk Between Inflammation and Cancer. Front. Immunol..

[230] Brabson J.P., Leesang T., Mohammad S., Cimmino L. (2021). Epigenetic Regulation of Genomic Stability by Vitamin C. Front. Genet..

[231] Wu J., Liang J., Li M., Lin M., Mai L., Huang X., Liang J., Hu Y., Huang Y. (2020). Modulation of miRNAs by vitamin C in H_2_O_2_-exposed human umbilical vein endothelial cells. Int. J. Mol. Med..

[232] Ferreira T.J., de Araújo C.C., da Silva Lima A.C., Matida L.M., Griebeler A.F.M., Coelho A.S.G., Gontijo A.P.M., Cominetti C., Vêncio E.F., Horst M.A. (2022). Dietary Intake is Associated with miR-31 and miR-375 Expression in Patients with Head and Neck Squamous Cell Carcinoma. Nutr. Cancer.

[233] Khajebishak Y., Alivand M., Faghfouri A.H., Moludi J., Payahoo L. (2023). The effects of vitamins and dietary pattern on epigenetic modification of non-communicable diseases: A review. Int. J. Vitam. Nutr. Res..

[234] Brown G. (2023). Retinoic acid receptor regulation of decision-making for cell differentiation. Front. Cell Dev. Biol..

[235] Bizzarri M., Giuliani A., Cucina A., Minini M. (2020). Redifferentiation therapeutic strategies in cancer. Drug Discov. Today.

[236] Lavudi K., Nuguri S.M., Olverson Z., Dhanabalan A.K., Patnaik S., Kokkanti R.R. (2023). Targeting the retinoic acid signaling pathway as a modern precision therapy against cancers. Front. Cell Dev. Biol..

[237] Takahashi N., Saito D., Hasegawa S., Yamasaki M., Imai M. (2022). Vitamin A in health care: Suppression of growth and induction of differentiation in cancer cells by vitamin A and its derivatives and their mechanisms of action. Pharmacol. Ther..

[238] Fujiki F., Morimoto S., Katsuhara A., Okuda A., Ogawa S., Ueda E., Miyazaki M., Isotani A., Ikawa M., Nishida S. (2022). T Cell-Intrinsic Vitamin A Metabolism and Its Signaling Are Targets for Memory T Cell-Based Cancer Immunotherapy. Front. Immunol..

[239] Caffrey A., Lamers Y., Murphy M.M., Letourneau N., Irwin R.E., Pentieva K., Ward M., Tan A., Rojas-Gómez A., Santos-Calderón L.A. (2023). Epigenetic effects of folate and related B vitamins on brain health throughout life: Scientific substantiation and translation of the evidence for health improvement strategies. Nutr. Bull..

[240] Franco C.N., Seabrook L.J., Nguyen S.T., Leonard J.T., Albrecht L.V. (2022). Simplifying the B Complex: How Vitamins B6 and B9 Modulate One Carbon Metabolism in Cancer and Beyond. Metabolites.

[241] Lionaki E., Ploumi C., Tavernarakis N. (2022). One-Carbon Metabolism: Pulling the Strings behind Aging and Neurodegeneration. Cells.

[242] Li X., Zhang H.-S. (2023). Amino acid metabolism, redox balance and epigenetic regulation in cancer. FEBS J..

[243] Yoo H.S., Shanmugalingam U., Smith P.D. (2022). Potential roles of branched-chain amino acids in neurodegeneration. Nutrition.

[244] Torres N., Tobón-Cornejo S., Velazquez-Villegas L.A., Noriega L.G., Alemán-Escondrillas G., Tovar A.R. (2023). Amino Acid Catabolism: An Overlooked Area of Metabolism. Nutrients.

[245] Maleknia M., Ahmadirad N., Golab F., Katebi Y., Haj Mohamad Ebrahim Ketabforoush A. (2023). DNA Methylation in Cancer: Epigenetic View of Dietary and Lifestyle Factors. Epigenet. Insights.

[246] Ilina A., Khavinson V., Linkova N., Petukhov M. (2022). Neuroepigenetic Mechanisms of Action of Ultrashort Peptides in Alzheimer’s Disease. Int. J. Mol. Sci..

[247] Janssens Y., Wynendaele E., Vanden Berghe W., De Spiegeleer B. (2019). Peptides as epigenetic modulators: Therapeutic implications. Clin. Epigenetics.

[248] Mukherjee A.G., Wanjari U.R., Gopalakrishnan A.V., Bradu P., Biswas A., Ganesan R., Renu K., Dey A., Vellingiri B., El Allali A. (2023). Evolving strategies and application of proteins and peptide therapeutics in cancer treatment. Biomed. Pharmacother..

[249] Azzaz F., Fantini J. (2022). The epigenetic dimension of protein structure. Biomol. Concepts.

[250] Santa-María C., López-Enríquez S., Montserrat-de la Paz S., Geniz I., Reyes-Quiroz M.E., Moreno M., Palomares F., Sobrino F., Alba G. (2023). Update on Anti-Inflammatory Molecular Mechanisms Induced by Oleic Acid. Nutrients.

[251] Choi S.-W., Friso S. (2023). Modulation of DNA methylation by one-carbon metabolism: A milestone for healthy aging. Nutr. Res. Pract..

[252] Korsmo H.W., Jiang X. (2021). One carbon metabolism and early development: A diet-dependent destiny. Trends Endocrinol. Metab..

[253] Bokor S., Vass R.A., Funke S., Ertl T., Molnár D. (2022). Epigenetic Effect of Maternal Methyl-Group Donor Intake on Offspring’s Health and Disease. Life.

[254] Genchi G., Lauria G., Catalano A., Sinicropi M.S., Carocci A. (2023). Biological Activity of Selenium and Its Impact on Human Health. Int. J. Mol. Sci..

[255] Alehagen U., Opstad T.B., Alexander J., Larsson A., Aaseth J. (2021). Impact of selenium on biomarkers and clinical aspects related to ageing. A review. Biomolecules.

[256] Zhang Y., He Q. (2022). The role of SELENBP1 and its epigenetic regulation in carcinogenic progression. Front. Genet..

[257] Huang X., Dong Y.L., Li T., Xiong W., Zhang X., Wang P.J., Huang J.Q. (2021). Dietary selenium regulates micrornas in metabolic disease: Recent progress. Nutrients.

[258] Babaeenezhad E., Naghibalhossaini F., Rajabibazl M., Jangravi Z., Hadipour Moradi F., Fattahi M.D., Hoheisel J.D., Sarabi M.M., Shahryarhesami S. (2022). The Roles of microRNA miR-185 in Digestive Tract Cancers. Non-Coding RNA.

[259] Pordel S., Khorrami M., Saadatpour F., Rezaee D., Cho W.C., Jahani S., Aghaei-Zarch S.M., Hashemi E., Najafi S. (2023). The role of microRNA-185 in the pathogenesis of human diseases: A focus on cancer. Pathol. Res. Pract..

[260] Qin X., Zhang X., Li P., Wang M., Yan L., Pan P., Zhang H., Hong X., Liu M., Bao Z. (2021). MicroRNA-185 activates PI3K/AKT signalling pathway to alleviate dopaminergic neuron damage via targeting IGF1 in Parkinson’s disease. J. Drug Target..

[261] Rahimmi A., Peluso I., Rajabi A., Hassanzadeh K. (2019). miR-185 and SEPT5 Genes May Contribute to Parkinson’s Disease Pathophysiology. Oxidative Med. Cell. Longev..

[262] Brito S., Lee M.-G.G., Bin B.-H.H., Lee J.-S.S. (2020). Zinc and its transporters in epigenetics. Mol. Cells.

[263] Sanusi K.O., Ibrahim K.G., Abubakar B., Malami I., Bello M.B., Imam M.U., Abubakar M.B. (2021). Effect of maternal zinc deficiency on offspring health: The epigenetic impact. J. Trace Elem. Med. Biol..

[264] Yusuf A.P., Abubakar M.B., Malami I., Ibrahim K.G., Abubakar B., Bello M.B., Qusty N., Elazab S.T., Imam M.U., Alexiou A. (2021). Zinc Metalloproteins in Epigenetics and Their Crosstalk. Life.

[265] Balaji E.V., Kumar N., Satarker S., Nampoothiri M. (2020). Zinc as a plausible epigenetic modulator of glioblastoma multiforme. Eur. J. Pharmacol..

[266] Zhao J., Wen D., Zhang S., Jiang H., Di X. (2023). The role of zinc finger proteins in malignant tumors. FASEB J..

[267] Sun R., Wang J., Feng J., Cao B. (2022). Zinc in Cognitive Impairment and Aging. Biomolecules.

[268] Neja S.A. (2020). Site-Specific DNA Demethylation as a Potential Target for Cancer Epigenetic Therapy. Epigenet. Insights.

[269] Jin J., Guo Y., Dong X., Liu J., He Y. (2020). Methylation-associated silencing of miR-193b improves the radiotherapy sensitivity of esophageal cancer cells by targeting cyclin D1 in areas with zinc deficiency. Radiother. Oncol..

[270] Jin J., Guo T., Guo Y., Liu J., Qu F., He Y. (2019). Methylation-associated silencing of miR-128 promotes the development of esophageal cancer by targeting COX-2 in areas with a high incidence of esophageal cancer. Int. J. Oncol..

[271] Yu H., Peng Y., Wu Z., Wang M., Jiang X. (2022). MiR-193b as an effective biomarker in human cancer prognosis for Asian patients: A meta-analysis. Transl. Cancer Res..

[272] Lanza M., Cuzzocrea S., Oddo S., Esposito E., Casili G. (2023). The Role of miR-128 in Neurodegenerative Diseases. Int. J. Mol. Sci..

[273] Budi H.S., Younus L.A., Lafta M.H., Parveen S., Mohammad H.J., Al-Qaim Z.H., Jawad M.A., Parra R.M.R., Mustafa Y.F., Alhachami F.R. (2023). The role of miR-128 in cancer development, prevention, drug resistance, and immunotherapy. Front. Oncol..

[274] Rossetti S., Sacchi N. (2020). Emerging cancer epigenetic mechanisms regulated by all-trans retinoic acid. Cancers.

[275] Lim J.Y., Wang X.-D.D. (2020). Mechanistic understanding of β-cryptoxanthin and lycopene in cancer prevention in animal models. Biochim. Biophys. Acta (BBA)-Mol. Cell Biol. Lipids.

[276] Xie K., Ngo S., Rong J., Sheppard A. (2019). Modulation of mitochondrial respiration underpins neuronal differentiation enhanced by lutein. Neural Regen. Res..

[277] Hao Y., Yi Q., XiaoWu X., WeiBo C., GuangChen Z., XueMin C. (2022). Acetyl-CoA: An interplay between metabolism and epigenetics in cancer. Front. Mol. Med..

[278] Zhang G., Zhang M., Pei Y., Qian K., Xie J., Huang Q., Liu S., Xue N., Zu Y., Wang H. (2023). Enhancing stability of liposomes using high molecular weight chitosan to promote antioxidative stress effects and lipid-lowering activity of encapsulated lutein in vivo and in vitro. Int. J. Biol. Macromol..

[279] Cui J., Li G., Yin J., Li L., Tan Y., Wei H., Liu B., Deng L., Tang J., Chen Y. (2020). GSTP1 and cancer: Expression, methylation, polymorphisms and signaling (Review). Int. J. Oncol..

[280] Vrânceanu M., Galimberti D., Banc R., Dragoş O., Cozma-Petruţ A., Hegheş S.-C.C., Voştinaru O., Cuciureanu M., Stroia C.M., Miere D. (2022). The Anticancer Potential of Plant-Derived Nutraceuticals via the Modulation of Gene Expression. Plants.

[281] Kubczak M., Szustka A., Rogalińska M. (2021). Molecular Targets of Natural Compounds with Anti-Cancer Properties. Int. J. Mol. Sci..

[282] Moody L., Crowder S.L., Fruge A.D., Locher J.L., Demark-Wahnefried W., Rogers L.Q., Delk-Licata A., Carroll W.R., Spencer S.A., Black M. (2020). Epigenetic stratification of head and neck cancer survivors reveals differences in lycopene levels, alcohol consumption, and methylation of immune regulatory genes. Clin. Epigenet..

[283] El Omari N., Bakha M., Imtara H., Guaouguaoua F.-E.E., Balahbib A., Zengin G., Bouyahya A. (2021). Anticancer Mechanisms of Phytochemical Compounds: Focusing on Epigenetic Targets. Environ. Sci. Pollut. Res..

[284] Peng C., Zeleznik O.A., Shutta K.H., Rosner B.A., Kraft P., Clish C.B., Stampfer M.J., Willett W.C., Tamimi R.M., Eliassen A.H. (2022). A Metabolomics Analysis of Circulating Carotenoids and Breast Cancer Risk. Cancer Epidemiol. Biomark. Prev..

[285] Rudzińska A., Juchaniuk P., Oberda J., Wiśniewska J., Wojdan W., Szklener K., Mańdziuk S. (2023). Phytochemicals in Cancer Treatment and Cancer Prevention—Review on Epidemiological Data and Clinical Trials. Nutrients.

[286] Vrdoljak N. (2022). Carotenoids and Carcinogenesis: Exploring the Antioxidant and Cell Signaling Roles of Carotenoids in the Prevention of Cancer. Crit. Rev. Oncog..

[287] Koklesova L., Liskova A., Samec M., Zhai K., Abotaleb M., Ashrafizadeh M., Brockmueller A., Shakibaei M., Biringer K., Bugos O. (2020). Carotenoids in Cancer Metastasis-Status Quo and Outlook. Biomolecules.

[288] Maghsoudi S., Taghavi Shahraki B., Rabiee N., Fatahi Y., Bagherzadeh M., Dinarvand R., Ahmadi S., Rabiee M., Tahriri M., Hamblin M.R. (2022). The colorful world of carotenoids: A profound insight on therapeutics and recent trends in nano delivery systems. Crit. Rev. Food Sci. Nutr..

[289] Saini R.K., Keum Y.-S.S., Daglia M., Rengasamy K.R. (2020). Dietary carotenoids in cancer chemoprevention and chemotherapy: A review of emerging evidence. Pharmacol. Res..

[290] Rowles J.L., Erdman J.W. (2020). Carotenoids and their role in cancer prevention. Biochim. Biophys. Acta (BBA)-Mol. Cell Biol. Lipids.

[291] Pandita D., Pandita A. (2022). Omics Technology for the Promotion of Nutraceuticals and Functional Foods. Front. Physiol..

[292] Jefrei E., Xu M., Moore J.B., Thorne J.L. (2021). A systematic scoping review of the molecular mechanisms underpinning phytosterol and phytostanol mediated epigenetic changes. Proc. Nutr. Soc..

[293] Pradhan N., Parbin S., Kar S., Das L., Kirtana R., Suma Seshadri G., Sengupta D., Deb M., Kausar C., Patra S.K. (2019). Epigenetic silencing of genes enhanced by collective role of reactive oxygen species and MAPK signaling downstream ERK/Snail axis: Ectopic application of hydrogen peroxide repress CDH1 gene by enhanced DNA methyltransferase activity in human breast cancer. Biochim. Biophys. Acta (BBA)-Mol. Basis Dis..

[294] Hassani B., Goshtasbi G., Nooraddini S., Firouzabadi N. (2022). Pharmacological Approaches to Decelerate Aging: A Promising Path. Oxidative Med. Cell. Longev..

[295] Yuan W., Fang W., Zhang R., Lyu H., Xiao S., Guo D., Ali D.W., Michalak M., Chen X.Z., Zhou C. (2023). Therapeutic strategies targeting AMPK-dependent autophagy in cancer cells. Biochim. Biophys. Acta (BBA)-Mol. Cell Res..

[296] Ares A.M., Tapia J.A., González-Porto A.V., Higes M., Martín-Hernández R., Bernal J. (2022). Glucosinolates as Markers of the Origin and Harvesting Period for Discrimination of Bee Pollen by UPLC-MS/MS. Foods.

[297] Shock T., Badang L., Ferguson B., Martinez-Guryn K. (2021). The interplay between diet, gut microbes, and host epigenetics in health and disease. J. Nutr. Biochem..

[298] Bouranis J.A., Beaver L.M., Ho E. (2021). Metabolic Fate of Dietary Glucosinolates and Their Metabolites: A Role for the Microbiome. Front. Nutr..

[299] Molinero N., Antón-Fernández A., Hernández F., Ávila J., Bartolomé B., Moreno-Arribas M.V. (2023). Gut Microbiota, an Additional Hallmark of Human Aging and Neurodegeneration. Neuroscience.

[300] Woo V., Alenghat T. (2022). Epigenetic regulation by gut microbiota. Gut Microbes.

[301] Celiker C., Kalkan R. (2020). Genetic and epigenetic perspective of microbiota. Appl. Microbiol. Biotechnol..

[302] Zhang X.-S.S., Yin Y.S., Wang J., Battaglia T., Krautkramer K., Li W.V., Li J., Brown M., Zhang M., Badri M.H. (2021). Maternal cecal microbiota transfer rescues early-life antibiotic-induced enhancement of type 1 diabetes in mice. Cell Host Microbe.

[303] Stein R.A., Riber L. (2023). Epigenetic effects of short-chain fatty acids from the large intestine on host cells. Microlife.

[304] Xiong R.-G., Zhou D.-D., Wu S.-X., Huang S.-Y., Saimaiti A., Yang Z.-J., Shang A., Zhao C.-N., Gan R.-Y., Li H.-B. (2022). Health Benefits and Side Effects of Short-Chain Fatty Acids. Foods.

[305] Fock E., Parnova R. (2023). Mechanisms of Blood-Brain Barrier Protection by Microbiota-Derived Short-Chain Fatty Acids. Cells.

[306] Chen H., Meng L., Shen L. (2022). Multiple roles of short-chain fatty acids in Alzheimer disease. Nutrition.

[307] Feitelson M.A., Arzumanyan A., Medhat A., Spector I. (2023). Short-chain fatty acids in cancer pathogenesis. Cancer Metastasis Rev..

[308] Xi Y., Jing Z., Wei W., Chun Z., Quan Q., Qing Z., Jiamin X., Shuwen H. (2021). Inhibitory effect of sodium butyrate on colorectal cancer cells and construction of the related molecular network. BMC Cancer.

[309] Urzì O., Gasparro R., Rabienezhad Ganji N., Alessandro R., Raimondo S., Ganji N.R., Alessandro R., Raimondo S. (2022). Plant-RNA in Extracellular Vesicles: The Secret of Cross-Kingdom Communication. Membranes.

[310] Li D., Yao X., Yue J., Fang Y., Cao G., Midgley A.C., Nishinari K., Yang Y. (2022). Advances in Bioactivity of MicroRNAs of Plant-Derived Exosome-Like Nanoparticles and Milk-Derived Extracellular Vesicles. J. Agric. Food Chem..

[311] Kadriya A., Falah M. (2023). Nanoscale Phytosomes as an Emerging Modality for Cancer Therapy. Cells.

[312] Xu X.H., Yuan T.J., Dad H.A., Shi M.Y., Huang Y.Y., Jiang Z.H., Peng L.H. (2021). Plant Exosomes As Novel Nanoplatforms for MicroRNA Transfer Stimulate Neural Differentiation of Stem Cells in Vitro and in Vivo. Nano Lett..

[313] Ishida T., Kawada K., Jobu K., Morisawa S., Kawazoe T., Nishimura S., Akagaki K., Yoshioka S., Miyamura M. (2023). Exosome-like nanoparticles derived from Allium tuberosum prevent neuroinflammation in microglia-like cells. J. Pharm. Pharmacol..

[314] Weng X., Wang H. (2022). Apical vesicles: Social networking at the pollen tube tip. Reprod. Breed..

[315] Cai Q., He B., Wang S., Fletcher S., Niu D., Mitter N., Birch P.R.J., Jin H. (2021). Message in a Bubble: Shuttling Small RNAs and Proteins between Cells and Interacting Organisms Using Extracellular Vesicles. Annu. Rev. Plant Biol..

[316] Saadeldin I.M., Tanga B.M., Bang S., Maigoro A.Y., Kang H., Cha D., Lee S., Lee S., Cho J. (2023). MicroRNA profiling of royal jelly extracellular vesicles and their potential role in cell viability and reversing cell apoptosis. Funct. Integr. Genom..

[317] Schuh C.M.A.P., Aguayo S., Zavala G., Khoury M. (2019). Exosome-like vesicles in Apis mellifera bee pollen, honey and royal jelly contribute to their antibacterial and pro-regenerative activity. J. Exp. Biol..

[318] Li Y., Pollock C.A., Saad S. (2021). Aberrant DNA Methylation Mediates the Transgenerational Risk of Metabolic and Chronic Disease Due to Maternal Obesity and Overnutrition. Genes.

[319] Natale F., Spinelli M., Rinaudo M., Cocco S., Nifo Sarrapochiello I., Fusco S., Grassi C. (2023). Maternal High Fat Diet Anticipates the AD-like Phenotype in 3xTg-AD Mice by Epigenetic Dysregulation of Aβ Metabolism. Cells.

[320] Reichetzeder C. (2021). Overweight and obesity in pregnancy: Their impact on epigenetics. Eur. J. Clin. Nutr..

[321] Cirulli F., Musillo C., Berry A. (2020). Maternal Obesity as a Risk Factor for Brain Development and Mental Health in the Offspring. Neuroscience.

[322] Phang M., Ross J., Raythatha J.H., Dissanayake H.U., McMullan R.L., Kong Y., Hyett J., Gordon A., Molloy P., Skilton M.R. (2020). Epigenetic aging in newborns: Role of maternal diet. Am. J. Clin. Nutr..

[323] Zaidan H., Wnuk A., Aderka I.M., Kajta M., Gaisler-Salomon I. (2023). Pre-reproductive stress in adolescent female rats alters maternal care and DNA methylation patterns across generations. Stress.

[324] Ando C., Ma S., Miyoshi M., Furukawa K., Li X., Jia H., Kato H. (2023). Postnatal nutrition environment reprograms renal DNA methylation patterns in offspring of maternal protein-restricted stroke-prone spontaneously hypertensive rats. Front. Nutr..

[325] Barreto S.G., Pandol S.J. (2021). Young-Onset Carcinogenesis—The Potential Impact of Perinatal and Early Life Metabolic Influences on the Epigenome. Front. Oncol..

[326] Ambeskovic M., Roseboom T.J., Metz G.A.S.S. (2020). Transgenerational effects of early environmental insults on aging and disease incidence. Neurosci. Biobehav. Rev..

[327] Comas-Armangue G., Makharadze L., Gomez-Velazquez M., Teperino R. (2022). The Legacy of Parental Obesity: Mechanisms of Non-Genetic Transmission and Reversibility. Biomedicines.

